# Natural metabolites targeting macrophage polarization for non-small cell lung cancer therapy: a review of mechanisms and applications based on chemical classification

**DOI:** 10.3389/fphar.2026.1856152

**Published:** 2026-09-25

**Authors:** Yuxuan Zhang, Lu Liu, Xiaoyan Dai, Hongyan Yan, Xudong Lei, Hua Yang

**Affiliations:** 1 Department of Pharmacy, Sun Yat-sen University Cancer Center Gansu Hospital, Lanzhou, Gansu, China; 2 Department of Traditional Chinese Medicine Management, Sun Yat-sen University Cancer Center Gansu Hospital, Lanzhou, Gansu, China; 3 School of Traditional Chinese and Western Medicine, Gansu University of Traditional Chinese Medicine, Lanzhou, Gansu, China

**Keywords:** macrophage polarization, natural metabolites, non-small cell lung cancer, tumor microenvironment, tumor-associated macrophages

## Abstract

Non-small cell lung cancer (NSCLC) accounts for roughly 85% of lung cancer cases and is a leading cause of cancer-related death worldwide. Reprogramming tumor-associated macrophages (TAMs) from a pro-tumor M2 phenotype to an anti-tumor M1 phenotype has emerged as a promising therapeutic strategy. This review examines naturally occurring metabolites that modulate TAM polarization in NSCLC, organized into eight chemical classes: flavonoids, alkaloids, terpenoids, polysaccharides, saponins, stilbenoids, lignans, and phenolic acids. A recurring finding is that these compounds converge on shared signaling pathways, particularly PI3K/Akt/mTOR, NF-κB, and STAT family members, while each class also displays distinct mechanistic tendencies. Flavonoids couple redox modulation with immune effects. Alkaloids engage multiple targets but carry dose-dependent toxicity. Terpenoids show strong tumor microenvironment penetration and synergy with immune checkpoint inhibitors. Polysaccharides act through membrane receptors such as Toll-like receptors and Dectin-1. Saponins largely center on AMPK signaling. The remaining classes, though supported by fewer studies, point to MDSC regulation and context-dependent effects. Multi-component plant extracts and traditional Chinese medicine formulas also influence TAM polarization and have reached adjuvant clinical use. Delivery platforms such as nanoparticles, hydrogels, and exosomes are being developed to address poor bioavailability. Translation remains limited by a lack of trials with TAM-specific endpoints, an oversimplified M1/M2 framework, and the need for standardized pharmacokinetic and targeting assessments. Future progress will require biopsy-based TAM phenotyping, biomarker-driven patient stratification, and delivery systems matched to metabolite properties.

## 1 Introduction

Non-small cell lung cancer (NSCLC) accounts for roughly 85% of lung cancer cases and causes approximately 1.8 million new cases and 1.6 million deaths annually (Bray et al., 2024). Despite chemotherapy, epidermal growth factor receptor (EGFR) inhibitors, and immune checkpoint blockers (ICBs) such as anti-programmed cell death protein 1 (PD-1)/programmed death-ligand 1 (PD-L1) antibodies, only 15%–20% of advanced patients respond to ICBs and most develop resistance (Chen and Mellman, 2013; Reck et al., 2016). Within the tumor microenvironment (TME), tumor-associated macrophages (TAMs) constitute 30%–50% of stromal cells, and their function depends on polarization state (Zhou et al., 2022). M1 macrophages drive antitumor immunity *via* interferon-γ (IFN-γ), tumor necrosis factor-α (TNF-α), interleukin-12 (IL-12), and markers such as CD80, CD86, and inducible nitric oxide synthase (iNOS), whereas M2 macrophages, dominant in advanced disease, promote angiogenesis, epithelial-mesenchymal transition (EMT), and drug resistance through IL-10, transforming growth factor-β (TGF-β), CD204, CD206, and arginase-1 (Mantovani et al., 2002; Qian and Pollard, 2010; Zhou et al., 2022). Because high M2 infiltration predicts poor prognosis, TAM polarization has become a key therapeutic target (Sumitomo et al., 2019), motivating the search for reprogramming agents, including natural metabolites.

Natural metabolites from plants, microorganisms, and traditional Chinese medicine (TCM) offer multi-target pharmacology, low toxicity, and holistic TME regulation (Deng et al., 2020; Maiuolo et al., 2021; Hu et al., 2023). Unlike single-pathway synthetic drugs, they can simultaneously modulate cytokines, signal transducer and activator of transcription 3 (STAT3), nuclear factor kappa-light-chain-enhancer of activated B cells (NF-κB), and cell-cell interactions, or deplete TAMs (Rajabi et al., 2021; Sui et al., 2020). Grouping these metabolites by chemical scaffold (flavonoids, alkaloids, terpenoids, *etc.*) organizes the literature and enables systematic comparison across structural classes. It is especially important to recognize, however, that the familiar M1/M2 dichotomy is an oversimplification. Single-cell RNA sequencing (scRNA-seq) studies have made it clear that TAMs do not exist as two discrete populations, but instead occupy a continuous spectrum that includes pro-inflammatory, immunosuppressive, angiogenic, and phagocytic functional states (Wu F. et al., 2021; Wang Y. et al., 2024). With this understanding, the M1 and M2 terminology is retained throughout this review as a convenient framework for summarizing how chemically classified natural metabolites modulate TAM polarization in NSCLC, for evaluating the supporting evidence, and for exploring possible connections between chemical structure and biological function that emerge when these metabolites are examined by class.

## 2 Literature search and study selection

This review was conducted in accordance with the Preferred Reporting Items for Systematic Reviews and Meta-Analyses (PRISMA) guidelines to ensure the transparency and reproducibility of literature retrieval and screening. A systematic literature search was performed across three biomedical databases, namely PubMed, the Cochrane Library, and Google Scholar, from database inception to 1 July 2026. The search strategy combined terms for NSCLC, TAMs, macrophage polarization, and natural metabolites using Boolean operators. The complete search strings for all databases are provided in Supplementary File 1. Two independent researchers performed the literature screening in duplicate, with discrepancies resolved through discussion with a third senior researcher. Studies were eligible for inclusion if they reported original experimental data on regulatory effects of natural metabolites, plant extracts, or traditional Chinese medicine formulations on TAM polarization in NSCLC models (cell lines, tumor-bearing animals, or clinical samples). After duplicate removal, 1,724 unique records underwent title and abstract screening, yielding 448 articles for full-text evaluation. Ultimately, 64 original studies met all inclusion criteria and were retained for this review. The PRISMA flow diagram is provided as Supplementary File 1 (Supplement Figure S1). All included metabolites were assessed against Pan-Assay Interference Compounds (PAINS) filters (Bolz et al., 2021). An evidence-level classification system was applied: Level A (clinical evidence for TAM polarization modulation), Level B (*in vivo* plus *in vitro* TAM-specific evidence), Level C (*in vitro* only), and Level D (indirect evidence). The detailed evidence classification table is provided as Supplementary Table S1.

## 3 Mechanisms of purified single-metabolite natural metabolites classified by chemical category

The role of natural metabolites in cancer therapy stretches back decades. Paclitaxel was isolated from the bark of the *Taxus brevifolia* Nutt., vinblastine and vincristine came from the *Catharanthus roseus* (L.) G.Don, camptothecin derivatives trace back to the tree *Camptotheca acuminata* Decne. Each of these molecules eventually became a first-line chemotherapy drug. Early screening efforts focused almost entirely on tumor cell proliferation and apoptosis. Over the past dozen or so years, immune cells in the TME have drawn increasing attention as targets, and macrophages, abundant in tumor tissue and functionally plastic, have moved to the center of that interest. Natural metabolites offer a rich compound library at exactly this point. Their structures are diverse, and long evolutionary selection has shaped them to engage multiple targets at once, a feature that fits reasonably well with a process like macrophage polarization, which is controlled by several signaling pathways simultaneously. A natural origin, of course, does not guarantee safety or efficacy. Many metabolites have poor bioavailability, and their mechanisms are not well understood. The sections that follow examine purified metabolites reported to influence TAM polarization in NSCLC models, organized by chemical class. The emphasis is on the signaling pathways they affect, the reliability of the model systems, and how far the evidence can go toward supporting translational work.

### 3.1 Flavonoids

Flavonoids are polyphenolic metabolites found in vegetables, fruits, grains, and tea leaves. Their C6-C3-C6 skeleton supports strong antioxidant activity and a range of other biological effects (Shen et al., 2022). The extended conjugation and multiple phenolic hydroxyl groups in this backbone allow them to scavenge radicals, chelate metals, and engage in redox cycling, making them particularly responsive signaling pathways sensitive to oxidative stress. Because they are potent, widely available, and easy to obtain, flavonoids have become a major focus in cancer prevention and treatment. They can be extracted from medicinal plants such as *Petroselinum crispum* (Mill.) Fuss, *Apium graveolens* L., and *Phyllanthus emblica* L., and they act through antioxidant, anti-inflammatory, and immune-regulating mechanisms (Hazafa et al., 2020). Their influence on TAMs polarization comes from targeted effects on pathways that control the M1/M2 balance, with few off-target actions (Dobrzynska et al., 2020; Patra et al., 2021; Tsai et al., 2021). In NSCLC, flavonoids also modulate immune responses, promote ROS production, and interact with miRNAs and proteins. When combined with anticancer drugs, radiotherapy, or nanoparticles, they can help reverse drug resistance and induce apoptosis in cancer cells (Liang M. S. et al., 2025; Wang X. et al., 2021).

**Baicalin** is isolated from the root of *Scutellaria baicalensis* Georgi, a traditional Chinese botanical drug with a millennia-long history of anti-inflammatory application (Fan et al., 2013; Gong et al., 2018). *In vitro* studies have revealed a dual regulatory mechanism for baicalin. These studies used RAW264.7 macrophages and THP-1-derived TAMs as models. First, baicalin directly inhibits the PI3K/Akt/mTOR pathway. This pathway drives M2 TAM polarization in NSCLC. Inhibition of this pathway upregulates M1 markers such as iNOS and CD86. It also increases pro-inflammatory cytokines including TNF-α and IL-12 (Fan et al., 2013; Wang et al., 2019). Second, baicalin reverses IL-4/IL-13-induced M2 polarization. It does so by reducing STAT6 phosphorylation. This effect lowers expression of M2 markers (arginase-1, CD206) and the immunosuppressive cytokine IL-10 (Fan et al., 2013). Together, these two pathways sustain an anti-tumor TAM phenotype. Beyond immunomodulation, baicalin also acts through redox-regulatory mechanisms. These effects have been demonstrated in A549 and A549/DDP NSCLC cell lines and xenograft mouse models. The study included groups treated with the NRF2 inducer baicalin and groups with KEAP1 knockout. Baicalin acts through the KEAP1-NRF2/HO-1 axis. It induces ferritin autophagy-associated proteins and autophagosome formation. It also promotes conversion of GSH to GSSG. This conversion facilitates M1 macrophage polarization. Combined treatment with cisplatin (DDP) amplifies these effects and increases NSCLC sensitivity to DDP. baicalin treatment and KEAP1 knockdown abolish the synergistic anti-tumor activity of baicalin plus DDP (Chen l. x. et al., 2024).

**Puerarin** is extracted from the root of *Pueraria montana* (Lour.) Merr. It is clinically used in China for cardiovascular disease treatment and cancer adjuvant therapy (Kang et al., 2017). Puerarin shows antitumor activity through multiple mechanisms. It inhibits cell migration and invasion, arrests the cell cycle, and promotes apoptosis (Hu et al., 2018; Huang et al., 2020). It also counteracts M2-type polarization by targeting the MEK/ERK1/2 pathway (Kang et al., 2017). Studies have tested puerarin in NSCLC xenograft models and IL-4-induced M2 macrophage models. *In vivo*, puerarin slows tumor growth. It raises levels of M1 markers such as CD197^+^ and pro-inflammatory factors such as IFN-γ. At the same time, it reduces M2 markers such as CD206^+^ and pro-tumor factors such as IL-10. *In vitro*, puerarin suppresses M2 macrophage polarization, NSCLC cell invasion and migration, and expression of angiogenesis factors. The underlying mechanism involves blocking ERK nuclear translocation. This action counteracts IL-4-induced polarization and metastasis (Kang et al., 2017).

**Quercetin**, a flavonoid widely distributed in fruits and vegetables, has documented anticancer, anti-inflammatory, and antiviral activities and also attenuates lipid peroxidation, platelet aggregation, and capillary permeability (Li et al., 2016). Its behavior when modulating macrophage polarization is notable for bidirectionality and environmental dependence, which distinguishes it from a generic anti-inflammatory agent. In non-tumor inflammatory models, quercetin favors an anti-inflammatory, pro-repair phenotype by activating the AMP-activated protein kinase (AMPK)/Akt signaling pathway and upregulating antioxidant enzymes and M2 markers (Tsai et al., 2021); under sterile inflammation, therefore, it reinforces an M2-skewed, resolution-oriented milieu. The functional orientation changes once a TME is introduced. In NSCLC and other malignancies, quercetin suppresses pro-tumor mediators such as secretory phosphoprotein 1 (SPP1) and C-X-C motif chemokine ligand 8 (CXCL8), blocks M2 polarization, and can reprogram TAMs toward an M1-like antitumor phenotype. Mechanistically, inhibition of macrophage-derived SPP1 downregulates M2 markers, including CD206 and arginase 1, and lowers anti-inflammatory cytokines such as IL-10 and TGF-β, while upregulating pro-inflammatory factors, among them IL-1β and TNF-α. These events coincide with reduced expression of cyclin-dependent kinase 1 (CDK1) and matrix metalloproteinase-2/9 (MMP-2/9), which impairs lung cancer cell proliferation, migration, and invasion (Wang B. et al., 2019). In the tumor setting, quercetin does not merely dampen inflammation but actively disrupts the immunosuppressive TAM niche. Studies using phorbol 12-myristate 13-acetate (PMA) plus IL-4-induced M2 TAMs, A549 NSCLC cells, and plasmid-transfected model systems have confirmed that quercetin-driven SPP1 suppression consistently blocks M2 polarization, as reflected by lower CD206, IL-10, and TGF-β alongside higher IL-1β and TNF-α, and reduces A549 cell growth, motility, and invasiveness through the downregulation of CDK1 and MMP-2/9 (Wu and He, 2025).

**Luteolin** is a naturally occurring metabolism present in vegetables, medicinal plants, and fruits; it demonstrates anti-cancer activity against multiple malignancies, including lung cancer (Imran et al., 2019a). In NSCLC cell lines, it inhibits TAM family receptor tyrosine kinases (RTKs) in a dose-dependent manner, leading to reduced Axl transcription and lower TAM protein levels. This effect overcomes cisplatin resistance in both parental and resistant A549/H460 cells (Lee et al., 2017). These data identify TAM/RTK/Axl signaling as a tractable node for restoring cisplatin sensitivity.

**Kaempferol** a flavonoid present in natural sources such as flowers, cabbage, and cauliflower, exhibits a range of pharmacological effects, including antibacterial, anti-inflammatory, antioxidant, and antitumor activities (Dong et al., 2025; Imran et al., 2019b). scRNA-seq analyses of kaempferol-treated lung adenocarcinoma (LUAD) specimens have uncovered considerable macrophage heterogeneity, with M2-like TAMs representing the predominant population. Complementary network pharmacology screening identifies targets linked to macrophage function, pointing to compounds that can selectively inhibit M2 polarization and correlate with key prognostic genes such as signal transducer and activator of STAT1, thereby offering translational potential for incorporating traditional Chinese medicine compounds into LUAD immunotherapy (Wan L. et al., 2025).

**Formononetin**, a phytoestrogen found in numerous legumes and clovers, it is one of the primary flavonoids in *Astragalus membranaceus* (Fisch.) Bunge (Aliya et al., 2023; Singh et al., 2024). In Lewis lung carcinoma-bearing mouse models, formononetin downregulates the transcriptional activity of the AMPK and wingless-type MMTV integration site family (Wnt) signaling pathways, an effect that increases the proportion of total macrophages and M1 macrophages while reducing the proportion of M2 macrophages, elevates the spleen-to-thymus ratio, and inhibits tumor growth and metastasis without significant hepatic or renal toxicity (Cai, 2024).

**Vitexin**, a dietary flavonoid widely present in foods and medicinal plants, has attracted increasing attention for its diverse pharmacological activities and favorable safety profile. It exhibits therapeutic potential across multiple biological systems through antioxidant, anti-inflammatory, anti-cancer, antibacterial and neuroprotective mechanisms (Lin J. et al., 2025; Peng et al., 2021; Yan W. et al., 2025). In lipopolysaccharide (LPS)/IL-4-induced RAW264.7 macrophage and A549 cell models, vitexin dose-dependently inhibits both M1 markers, including iNOS, IL-1β, and nitric oxide (NO), and M2 markers, such as arginase-1 (Arg-1), mannose receptor (MR), and CD206, by modulating the STAT signaling pathway, specifically through reducing p-STAT3 levels, thereby suppressing M2 macrophage polarization and attenuating A549 cell migration driven by M2 macrophages (Zhao B. et al., 2020).

**Isoginkgetin**, a biflavonoid isolated from *Ginkgo biloba* L. Leaves, possessing a broad range of pharmacological activities, including anti-inflammatory and anti-oxidative stress effects. It has shown significant efficacy in combating hypercholesterolemia, atherosclerosis (Zhang Z. et al., 2025), osteoarthritis (Xu M. et al., 2025), and depression (Li P. et al., 2021). Moreover, isoginkgetin acts as a potential CDK6 inhibitor, inducing cytotoxic autophagy in hepatocellular carcinoma cells (Yao et al., 2023), and synergizes with doxorubicin to promote autophagic cell death in hepatocellular carcinoma (Wang Y. et al., 2022). In NSCLC, isoginkgetin promotes M1 macrophage polarization and suppresses tumor growth through a distinctive oxidative stress mechanism: by directly binding to and downregulating tribbles pseudokinase 3 (TRIB3), isoginkgetin suppresses the nuclear factor erythroid 2-related factor 2 (Nrf2) antioxidant pathway, which in turn induces oxidative stress, inhibits tumor cell proliferation, and modifies the TME (Zhu et al., 2026).

The flavonoids reviewed above engage an array of mechanistically distinct routes. These routes shift the balance of TAMs away from a pro-tumor M2 phenotype in lung cancer models. These compounds do not converge on a single master pathway. They collectively demonstrate that TAM reprogramming can occur through kinase cascades, transcriptional regulators, and oxidative stress circuits. The polyphenolic scaffold is a common structural feature of this class. This scaffold appears to confer a unique redox-immune coupling property. Several flavonoids, such as baicalin and isoginkgetin, directly engage oxidative stress pathways including KEAP1/NRF2. This engagement enables these compounds to simultaneously modulate the redox milieu and the immune polarity of TAMs. This dual activity is less prominent in other chemical classes. This structural feature also entails inherent pharmacokinetic trade-offs. Their poor aqueous solubility and rapid glucuronidation require advanced formulation strategies to achieve therapeutic exposures. These compounds generally have favorable safety profiles. They also enhance chemotherapy responses. These properties reinforce their translational promise in NSCLC immunotherapy. Head-to-head comparisons with metabolites from other classes are still needed. Such comparisons will determine whether the redox-immune coupling confers a genuine *in vivo* advantage.

### 3.2 Alkaloids

Alkaloids have long been a productive source of drug leads. Several that were isolated from botanical sources, including camptothecin and vincristine, have become established clinical anticancer agents (Cai C. et al., 2024; Lu J. J. et al., 2012; Sun Y. et al., 2009). Their effects on TAM polarization arise from broad regulation of TLR signaling, NF-κB activation, and cytokine secretion (Li Y. et al., 2025; Premkumar et al., 2010; Yang C. et al., 2017), and they can also counteract tumor drug resistance (Zhao Z. et al., 2024). The nitrogen-containing heterocyclic core common to this class carries weak basicity, which helps the molecules form hydrogen bonds with acidic residues in kinase hinge regions. This feature allows them to engage multiple signaling nodes at once. That same basicity, however, also contributes to dose-dependent off-target effects and hepatotoxicity, so structural refinement and controlled delivery remain important for moving these compounds forward.

**Vinblastine**, an alkaloid extracted from the *C. roseus* (L.) G. Don, classified as a microtubule-associated inhibitor. It is clinically employed in the treatment of various cancers, including Hodgkin’s lymphoma, NSCLC, and bladder cancer (Haque et al., 2018; Sears and Boger, 2015). In M0, M1, and M2 macrophages, TAMs, and tumor-bearing mouse models, including macrophage/CD8^+^ T cell depletion and anti-PD-1 combination arms, vinblastine disrupts microtubule stability, activates NF-κB and cytochrome b-245 alpha chain (CYBA)-dependent reactive oxygen species (ROS) production, promotes the nuclear translocation of even-skipped homeobox (EB) and lysosomal biogenesis, drives M2-like TAMs toward an M1-like phenotype, and enhances macrophage phagocytic activity. Its antitumor efficacy depends on both macrophages and CD8^+^ T cells, supporting its potential as a partner for ICBs in malignant tumors (Wang Y. N. et al., 2023).

**Matrine**, a naturally occurring bioactive metabolite extracted from traditional Chinese botanical drugs such as *Sophora flavescens* Aiton, *Sophorae tonkinensis* Gagnep (Li X. et al., 2021). It exhibits a broad spectrum of pharmacological activities, including antitumor, anti-inflammatory, analgesic, antifibrotic, antiviral, and antiarrhythmic effects, and enhancement of immune function (Chen F. et al., 2022; Lin Y. et al., 2022). In a co-culture system of RAW264.7 macrophages and Lewis lung carcinoma cells, and in tumor-bearing mouse models, matrine inhibits M2-like TAM polarization through the PI3K/Akt/mTOR pathway, reducing CD206 and IL-4 without affecting M1-type TAMs. This M2 blockade impairs EMT by upregulating E-cadherin and downregulating N-cadherin, suppresses lung cancer metastasis *in vitro*, promotes CD4^+^ and CD8^+^ T cell proliferation, and inhibits T helper 2 (Th2) cells *in vivo* (Zhao B. et al., 2021). A newly isolated derivative, matrine A, exhibits enhanced cytotoxicity: in NSCLC cells and an A549 orthotopic model, it induces pyroptosis by activating the NOD-like receptor protein 3 (NLRP3)/caspase-1/gasdermin D (GSDMD) pathway, blocks ROS production and autophagy *via* PI3K/Akt/mTOR inhibition, and suppresses EMT and angiogenesis, thereby achieving multidimensional anticancer activity (Luo et al., 2023).

**Sophoridine**, a stereoisomer of matrine. Both alkaloids are extracted from *Sophora alopecuroides* L., and sophoridine is present at relatively low levels in *S. flavescens* Aiton, displays antitumor, anti-inflammatory, antiviral, antibacterial, analgesic, cardioprotective, and immunoprotective activities (Chen et al., 2023). Sophoridine has been shown to treat NSCLC through ROS-mediated pyroptosis and autophagy (Luo et al., 2023).

**Sanguinarine**, a benzopyridine alkaloid derived from *Sanguinaria canadensis* L. root, exhibiting antioxidant, anti-inflammatory, pro-apoptotic, and growth-inhibitory effects against multiple cancers (Fu C. et al., 2018). It demonstrates distinct anti-cancer activity in NSCLC (Qin et al., 2022). Sanguinarine engages the NF-κB axis through an exosome-mediated mechanism: in A549-THP-1 co-culture systems, A549-derived exosomes inhibit the NF-κB pathway in THP-1 cells, as reflected by downregulated p-p65 protein and reduced TNF-α, IL-6, and C-C motif chemokine ligand 2 (CCL-2) expression, thereby attenuating A549 cell invasion, proliferation, and migration (Yu Y. et al., 2020). In Lewis lung carcinoma models and myeloid-derived suppressor cell (MDSC) systems, sanguinarine further acts on NF-κB to induce MDSC apoptosis and differentiation into macrophages and dendritic cells, downregulating Arg-1, iNOS, and ROS, alleviating MDSC-mediated suppression of CD8^+^ T cell proliferation and ameliorating tumor-induced immunosuppression (Li B. B. et al., 2022).

**Cinobufagin**, a bufadienolide extracted from the dried skin of the Chinese giant toad (*Bufo gargarizans* Cantor) displays potent antitumor activity across several cancer types (Jia J. et al., 2022). In an IL-4-induced THP-1-derived M2 macrophage model, cinobufotalin reverses dimethyloxalylglycine (DMOG)-promoted glycolytic activation, immunosuppressive polarization, and elevated PD-L1 expression, reduces TGF-β and IL-10 secretion, and inhibits lung cancer cell migration and angiogenesis. The underlying mechanism likely involves enhanced hydroxylation and ubiquitin-mediated degradation of hypoxia-inducible factor-1α (HIF-1α), thereby targeting HIF-1α and reprogramming glycolysis within the TME (Sun S. et al., 2025).

**13-methyl-palmatrubine**, an alkaloid obtained from *Corydalis yanhusuo* W.T.Wang ex Z.Y.Su and C.Y.Wu extract (Cheng X. et al., 2008; Lu Z. et al., 2012), acts on IL-4- and IL-13-induced M2-TAM models, A549 and NCL-H1975 NSCLC cells (including co-culture with M2-TAM conditioned medium), and human umbilical vein endothelial cells (HUVECs) exposed to M2-TAM conditioned medium. It inhibits NSCLC cell proliferation, invasion, EMT, and *in vivo* growth while promoting apoptosis, induces a dose-dependent shift from an M2 to an M1 TAM phenotype, and suppresses M2-TAM-driven malignant behavior and angiogenesis. The mechanism involves inactivation of the PI3K/Akt and JAK/signal transducer and activator of STAT3 signaling pathways in both NSCLC cells and HUVECs (Wu Z. et al., 2022).

These alkaloids converge on attenuating the M2-oriented TAM niche and promoting antitumor immunity through mechanistically distinct targets. The frequent engagement of ROS and NF-κB circuits highlights their immunomodulatory breadth. The nitrogen-containing heterocyclic scaffold of alkaloids appears to enable this multi-node targeting capacity, as their weak basicity facilitates interactions with diverse kinase and transcription factor families. This broad reactivity, however, also underlies a defining translational constraint. Alkaloids exhibit dose-dependent toxicity, exemplified by the hepatotoxicity of high-dose matrine. Structural modifications aimed at improving the therapeutic index, such as matrine derivatives with reduced nitrogen reactivity, have demonstrated potential in preclinical studies (Chen Z. et al., 2024; Dai et al., 2023; Li X. et al., 2021; Wang Q. et al., 2022). Together with their compatibility with chemotherapy and immune checkpoint blockade, these properties position alkaloids as promising candidates for combination regimens in NSCLC. The mechanistic evidence for sophoridine and 13-methylpalmatrubine, however, remains limited to *in vitro* systems, and their TAM-modulating claims require further *in vivo* validation before clinical consideration.

### 3.3 Terpenoids

Terpenoids are built from isoprene units and represent the most abundant and structurally diverse family of plant-derived **metabolites** (Tholl, 2015). Paclitaxel, a well-known member of this class, is a broad-spectrum anticancer drug with high potency and relatively low toxicity, used in breast, ovarian, head and neck, and lung cancers (Zhu and Chen, 2019). The lipophilic character of terpenoids helps them penetrate the TME, where they can modulate oxidative stress and metabolic pathways tied to macrophage polarization (Raue et al., 2021; Zhao Y. et al., 2018). Their repeating isoprene backbone gives them a high hydrocarbon content and rigid structure, which together account for their efficient membrane permeability and tumor tissue penetration.

**Paclitaxel**, a natural metabolite obtained from *Taxus* spp. (e.g., *Taxus chinensis* (Pilg.) Rehd.), has been a cornerstone of treatment for lung, breast, and ovarian cancers since its approval by the U.S. Food and Drug Administration (FDA) in 1992 (Yu H. et al., 2025). Although its canonical mechanism centers on mitotic arrest, paclitaxel also remodels the TME by acting on TAMs, where it drives Toll-like receptor 4 (TLR4) signaling to activate type I and II interferon pathways (Swanton et al., 2007; Yu D. et al., 2022). Through the TLR4-MyD88-NF-κB axis, paclitaxel mobilizes antitumor immune events, shifts M2-type TAMs toward an M1 phenotype, and enhances cross-presentation of tumor antigens, thereby rendering immunologically “cold” tumors responsive to ICBs (Kalinski et al., 2025).

**Tanshinone I**, a diterpenoid isolated from *Salvia miltiorrhiza* Bunge, clinically employed in the treatment of cardiovascular diseases and as an adjuvant therapy for NSCLC (Lee C. et al., 2008; Tian et al., 2021). In CL1-5 lung adenocarcinoma cells stimulated by macrophage-conditioned medium and in severe combined immunodeficient (SCID) mouse xenograft models, tanshinone I exhibits no direct cytotoxicity, instead suppressing IL-8 transcription by inhibiting activator protein-1 (AP-1) and NF-κB DNA-binding activity, modulating Ras-mitogen-activated protein kinase (MAPK) and Rac1 pathways, and reducing gelatinase activity, which collectively impairs CL1-5 migration and invasion *in vitro* and decreases tumor formation and metastasis *in vivo* (Lee C. et al., 2008).

**Tanshinone IIA**, an abietane-type diterpenoid also from *S. miltiorrhiza* Bunge. It not only exhibits anticancer activity but also modulates tumor PD-L1 levels and may enhance the efficacy of PD-1 inhibitors (Zhang Y. Z. et al., 2024; Zhao J. Y. et al., 2022). In LPS-treated RAW264.7 macrophages, tanshinone IIA promotes M2 polarization by improving mitochondrial function, modulating the TLR4-high mobility group box 1 (HMGB1)/CCAAT/enhancer binding protein β (CEBP-β) pathway, suppressing miR-155, and upregulating Arg1, found in inflammatory zone 1 (FIZZ1), and CD206 while downregulating iNOS and IL-1β (Gao S. et al., 2019). In an LPS-induced mouse model of acute lung injury (ALI), it similarly increases the M2 subtype, reduces the M1 subtype, inhibits NF-κB and HIF pathways, mitigates cytokine storms and inflammatory damage, and enhances alveolar epithelial repair (Zhao J. et al., 2022).

**Dihydrotanshinone I**, another diterpenoid constituent of *S. miltiorrhiza* Bunge, exhibiting anticancer, cardiovascular protective, and anti-inflammatory activities (Chen X. et al., 2019). Dihydrotanshinone I directly target and inhibits phosphoglycerate dehydrogenase (PHGDH) in tumor cells, blocking the serine biosynthesis pathway and reducing S-adenosyl methionine (SAM) production, which subsequently decreases histone H3 lysine four trimethylation (H3K4me3) levels, suppresses IL-10 and TGF-β transcription, and ultimately prevents macrophage polarization toward the M2 phenotype. Conditioned medium experiments confirmed that medium from Dihydrotanshinone I-treated Lewis lung carcinoma (LLC) cells significantly reduced Cd206 mRNA expression in bone marrow-derived macrophages (BMDMs), and scRNA-seq further revealed that M2-like macrophage clusters and the Arg1^+^ triggering receptor expressed on myeloid cells 2 (TREM2)^+^ regulatory macrophage subpopulation were markedly diminished. Flow cytometry validated an increased proportion of F4/80^+^CD86^+^ (M1) macrophages and a decreased proportion of F4/80^+^CD206^+^ (M2) macrophages. Combination with an anti-PD-1 antibody produced a significant synergistic effect that enhanced CD8^+^ T cell antitumor function, while depletion of CD8a^+^ T cells partially reversed the antitumor activity (Qian C. et al., 2026).

**Cryptotanshinone**, a lipophilic diterpenoid from *S. miltiorrhiza* Bunge, exhibiting potent anti-inflammatory and anti-tumor activity. It induces apoptosis in cancer cells and, when combined with drug therapy, inhibits cell proliferation, migration, and invasion (Han Z. et al., 2019; Wu Y. et al., 2020). Although direct evidence for cryptotanshinone modulating macrophage polarization in NSCLC is lacking, studies in Hepa1-6 hepatocellular carcinoma cells, mouse BMDMs, and hepatocellular carcinoma models show that it induces apoptosis *via* the JAK2/signal transducer and activator of STAT3 pathway and polarizes BMDMs toward the M1 phenotype through the TLR7/MyD88/NF-κB pathway, and *in vivo* it activates tumor-infiltrating immune cells and induces CD8^+^ T cell-dependent antitumor T-cell responses that, combined with anti-PD-L1 therapy, yield long-term specific immunity (Han Z. et al., 2019).

**Artesunate**, a semisynthetic artemisinin derivative from *Artemisia annua* L., induces apoptosis and autophagy, influences the TME, modulates immune responses, and overcomes drug resistance (Zhang J. et al., 2022; Fan X. et al., 2024). In LLC mouse models and *in vitro* macrophage and T cell systems, artesunate inhibits LLC progression, enhances T cell infiltration, reprograms TAMs toward an inflammatory phenotype, slows T cell exhaustion, and synergizes with anti-PD-1 therapy. Mechanistically, it promotes macrophage reprogramming through STAT1/interferon regulatory factor 1 (IRF1)-mediated M1 repolarization, upregulating genes linked to phagocytosis and antigen processing/presentation, with interferon signaling acting as a key pathway (Hu et al., 2025).

**Betulinic acid**, a pentacyclic triterpenoid obtained from *Betula* species such as *Betula platyphylla* Suk. and accessible through chemical synthesis and biotransformation, enhances chemosensitivity by inducing mitochondrial oxidative stress and regulating transcription factors (Aswathy et al., 2022; Jiang et al., 2021). In H1299, A549, and LLC cells and an LLC subcutaneous tumor model, betulinic acid inhibits NSCLC cell viability and clonogenicity, induces mitochondrial apoptosis, reduces MMP-2 expression to block EMT, and decreases the proportion of CD206^+^ M2 macrophages. *In vivo*, it retards tumor growth and increases the M1/M2 ratio by inhibiting the mTOR signaling pathway to repolarize TAMs (Zeng et al., 2024).

**Triptolide**, a bioactive metabolite from *Tripterygium wilfordii* Hook. f., acts on macrophages in multiple disease models, modulating migration, polarization, phagocytosis, and cytokine/chemokine production through various signaling pathways (Chen Y. et al., 2025). In an azoxymethane (AOM)/dextran sulfate sodium (DSS)-induced co-inoculation model involving ICR-1 mice, 4T1 cells, and M2-polarized RAW264.7 cells, triptolide inhibits macrophage differentiation toward the M2 phenotype, downregulates CD206, arginase 1, CD204, and anti-inflammatory cytokine secretion, and consequently eliminates M2 macrophage-mediated tumor progression (Li H. et al., 2020).

**β-Elemene**, a sesquiterpenoid from *A. annua* L., inhibits proliferation, promotes apoptosis, suppresses migration and invasion, and ameliorates the immune microenvironment, serving as an adjuvant against melanoma, glioblastoma, and prostate cancer (Feng Y. et al., 2024). In A549 subcutaneous xenograft models and A549/H1299 macrophage co-culture systems, β-elemene actively drives macrophage M1 polarization through an exosome-mediated miRNA delivery mechanism. It inhibits chromobox homolog 8 (CBX8) in tumor cells, promoting nuclear export of precursor-miR-127 and maturation of miR-127-3p, while upregulating synaptotagmin-binding cytoplasmic RNA-interacting protein (SYNCRIP) to facilitate miR-127-3p packaging into tumor-derived exosomes. Upon macrophage uptake, exosomal miR-127-3p targets and suppresses zinc finger CCCH-type containing 4 (ZC3H4), a negative regulator of M1 polarization, thereby upregulating CD86, iNOS, IL-1β, and TNF-α (Wu X. et al., 2024).

**Scutebarbatine A,** a diterpenoid alkaloid from *Scutellaria barbata* D. Don, has demonstrated anti-inflammatory and antitumor activities (Hao X. et al., 2023). In NSCLC cell and macrophage-conditioned medium co-culture systems and nude mouse xenograft models, scutebarbatine A suppresses the PI3K/Akt pathway and downregulates CD206, Arg-1, and peroxisome proliferator-activated receptor γ (PPAR-γ) expression, thereby reversing M2 macrophage-mediated promotion of NSCLC progression and inhibiting tumor growth *in vivo* (Zhang Y. et al., 2026).

**Tubeimoside II**, a triterpenoid saponin from *Bolbostemma paniculatum* (Maxim.) Franquet, carries a C-16 hydroxyl group that confers superior anticancer activity and lower toxicity relative to tubeimoside I (Liu X. et al., 2026). In NSCLC xenograft models, lung metastasis models, and patient-derived xenograft (PDX) models, tubeimoside II exerts synergistic ferroptosis-immune remodeling through a dual-target mechanism involving HERC2 and JAK2. Multi-omics analyses and CRISPR-Cas9 functional validation revealed that tubeimoside II binds HERC2 and impairs the ubiquitination and degradation of nuclear receptor coactivator 4 (NCOA4), inducing ferritinophagy-dependent ferroptosis with increased ROS, lipid peroxidation, and ferrous iron levels. Simultaneously, it targets JAK2 to suppress the JAK2/STAT3 pathway, promoting M1-like TAM polarization and dendritic cell maturation, enhancing CD8^+^ and CD4^+^ T cell IFN-γ^+^ cytotoxic T lymphocyte (CTL) proportions, and reducing regulatory T cells, MDSCs, and M2-like macrophages. A positive feedback loop links ferroptosis and immunity: ferroptosis releases damage-associated molecular patterns (DAMPs) that activate innate immunity, and activated CD8^+^ T cells secrete IFN-γ to further enhance ferroptosis through acyl-CoA synthetase long-chain family member 4 (ACSL4)-mediated lipid peroxidation. Combination with anti-PD-1 produced the strongest antitumor effects in subcutaneous and metastasis models (Xu T. et al., 2026).

Terpenoids are structurally diverse and reprogram TAMs through multiple routes. Some disrupt microtubules or activate TLR4, while others block serine synthesis indirectly, deliver exosomal miRNAs, or drive transcriptional reprogramming *via* STAT1/IRF1. mTOR inhibition and dual ferroptosis-immune induction add further layers. Functionally, these compounds steer macrophages toward an M1-like phenotype and strengthen CD8^+^ T cell activity. Their consistent synergy with anti-PD-1/PD-L1 therapy makes them attractive partners for combination regimens in NSCLC. The isoprene-derived hydrocarbon backbone makes terpenoids highly lipophilic. This aids penetration into the tumor microenvironment and engagement of intracellular targets, but it also creates formulation challenges. Poor aqueous solubility and rapid clearance mean that sophisticated delivery systems, such as albumin nanoparticles, exosomal carriers, and polymeric micelles, are often needed to reach therapeutic exposures.

Different compounds illustrate the range of mechanisms at play. Context shapes outcome: tanshinone IIA promotes M2 polarization in acute inflammatory settings, a reminder that the same molecule can behave differently depending on the microenvironment. Compound-specific toxicities also need rigorous evaluation. Cryptotanshinone provides indirect evidence of TAM repolarization in liver cancer models, but its effects in NSCLC await direct experimental confirmation.

### 3.4 Polysaccharides

Polysaccharides are carbohydrate polymers obtained from traditional Chinese botanical drugs such as Astragalus membranaceus and Dendrobium officinale. They are known for broad immunomodulatory activity. Their anticancer effects involve direct cytotoxicity as well as inhibition of M2-TAM-driven tumor promotion, largely through TLR-dependent M1 polarization and expansion of M1 macrophages (Yarley et al., 2021; Zhou et al., 2022). Because polysaccharides are large and hydrophilic, they cannot passively cross cell membranes. Their biological activity depends on recognition by surface receptors like TLRs and Dectin-1 rather than on intracellular entry. This receptor-mediated mode of action gives them a broad innate immune activation profile and generally low systemic toxicity.

**Astragalus Polysaccharides**, isolated from *A. membranaceus* (Fisch.) Bunge, exhibit antitumor and immunomodulatory properties, regulating immune cell function and improving the tumor immune microenvironment (Li C. X. et al., 2022; Qin Z. et al., 2025). Astragalus Polysaccharides significantly upregulates the M1/M2 macrophage polarization ratio in H441 and H1299 cells, *ex vivo* systems of clinical lung cancer samples, and NSCLC mouse models, while downregulating pro-tumor factors such as IL-6 and IL-10 in conditioned media. Astragalus Polysaccharides also promotes the functional maturation of dendritic cells (DCs) and enhances T cell-mediated anticancer immunity, inhibits NSCLC cell proliferation, clonogenic formation, and tumor spheroid growth *in vitro*, and produces time-dependent depletion of M2 macrophages *in vivo*, synergizing with cisplatin to strengthen anticancer efficacy while mitigating adverse reactions (Bamodu et al., 2019).

**β-Glucan**, a glucan derived from oats, barley, wheat, baker’s yeast, fungal cell walls, and certain microorganisms, possesses antioxidant and antitumor effects (Akramiene et al., 2007; Ciecierska et al., 2019). Acting on *in vitro* macrophage systems and tumor-bearing mouse models that include macrophage depletion and mixed injection of TAMs with tumor cells, β-glucan engages a Dectin-1 (C-type lectin domain family 7 member A)–spleen tyrosine kinase (Syk)–caspase recruitment domain family member 9 (CARD9)–extracellular signal-regulated kinase (ERK) pathway to convert immunosuppressive TAMs and alternatively activated macrophages to a classically activated phenotype, a shift that is accompanied by metabolic reprogramming characterized by enhanced glycolysis, Krebs cycle activity, and glutamine utilization. Oral administration delays tumor growth, promotes TAM phenotypic conversion and effector T cell activation, and reduces tumor burden and angiogenesis, with macrophage depletion substantially diminishing its efficacy (Liu et al., 2015).

**Lentinan**, a polysaccharide extracted from *Lentinus edodes* (Berk.) Sing., stimulates immune cells through pathways such as TLR4/Dectin-1/MAPK and Syk–protein kinase C (PKC)/NF-κB (Chihara et al., 1987; Zhang M. et al., 2019). When combined with chemotherapy, lentinan improves therapeutic efficacy in lung cancer while reducing the incidence and severity of adverse reactions (Zhang Y. et al., 2018; Zhao C. et al., 2021). In NSCLC cells and TAM models, lentinan does not affect tumor cell viability but inhibits invasion, lowers MMP9 expression, and attenuates M2 polarization in TAMs, possibly through suppression of Wnt/β-catenin signaling (Yang Y. et al., 2024). A meta-analysis of 1390 NSCLC patients receiving lentinan plus cisplatin pleural injection reported significantly superior clinical efficacy (relative risk [RR] 1.34), response rate (RR 1.51), and quality of life (RR 1.48) compared with cisplatin alone, along with fewer adverse events, although these findings await validation from high-quality randomized controlled trials (Zhao C. et al., 2021).

These three polysaccharides regulate TAM polarization through membrane receptor engagement rather than intracellular signaling. Astragalus polysaccharides upregulate the M1/M2 ratio in NSCLC models while promoting dendritic cell maturation and enhancing T cell-mediated immunity. β-Glucan acts through the Dectin-1/Syk/CARD9/ERK pathway to convert immunosuppressive TAMs to a classically activated phenotype, accompanied by metabolic reprogramming. Lentinan suppresses M2 polarization *via* Wnt/β-catenin inhibition and reduces NSCLC invasion *in vitro*. The high molecular weight and hydrophilicity of polysaccharides dictate their receptor-dependent mechanism. These compounds cannot cross cell membranes passively. Their biological activity depends entirely on surface receptor engagement, primarily TLRs and Dectin-1. This property confers broad innate immune activation with generally low systemic toxicity. But the limited number of compounds in this class and the absence of head-to-head comparisons prevent reliable structure-activity relationship inferences.

### 3.5 Saponins

Saponins are found widely in plants and fungi and display a broad range of activities, including hypoglycemic, hepatoprotective, anti-inflammatory, antioxidant, and anticancer effects (Güçlü-Ustündağ and Mazza, 2007; Li Y. et al., 2023). In lung cancer, saponin-containing metabolites can enhance M1 macrophage polarization and reduce inflammation (Zhou et al., 2022). Their amphiphilic structure lets them interact with cholesterol in cell membranes, affecting membrane fluidity and receptor signaling. The aglycone portion enables membrane penetration and engagement of intracellular targets, while the sugar chains modulate solubility and recognition by membrane transporters.

#### 3.5.1 Representative metabolites

**Ginsenoside Rh2** derived from *Panax ginseng* C.A.Mey. root, possesses anti-inflammatory, anti-allergic, anti-depressant, and anticancer activities (Guan and Qi, 2023). It inhibits tumor proliferation and migration, induces cell cycle arrest and programmed cell death, suppresses metastasis, mitigates drug resistance, and modulates the immune system (He X. et al., 2022; Liu et al., 2024b; Sun and Cheng, 2022). In RAW264.7 and THP-1 derived macrophages, A549 and H1299 NSCLC cells, and M2 macrophage–NSCLC co-culture systems, ginsenoside Rh2 converts the M2 phenotype to an M1 phenotype, downregulates vascular endothelial growth factor (VEGF), MMP-2, and MMP-9 at both mRNA and protein levels, reduces VEGF release, and inhibits M2 macrophage-driven NSCLC cell migration, with corresponding reductions in CD206 and VEGF observed *in vivo* (Li H. et al., 2018). A subsequent study integrating GEO dataset analysis, network pharmacology, molecular docking, and experimental validation identified Aurora kinase A (AURKA) as the core target through which ginsenoside Rh2 suppresses NSCLC. By downregulating AURKA, ginsenoside Rh2 lowers HIF-1α expression and reduces glucose consumption, lactate production, and ATP levels, thereby inhibiting tumor cell glycolysis. In PMA-induced THP-1 macrophage and NSCLC co-cultures, ginsenoside Rh2 increased the proportion of iNOS-positive M1 macrophages and decreased CD206-positive M2 macrophages, effects that were reversed by AURKA overexpression. Conditioned medium experiments further confirmed that medium from ginsenoside Rh2-treated tumor cells suppressed NSCLC colony formation, migration, and glycolysis, whereas medium from the AURKA overexpression group lacked such activity. The combination of ginsenoside Rh2 with cisplatin produced synergistic enhancement of apoptosis both *in vitro* and *in vivo*, a synergy that was abolished by AURKA overexpression (Chen Y. et al., 2026).

**Ginsenoside Re**, obtained from the fruit, leaves, stems, flower buds, and roots of ginseng as well as other Araliaceae plants, has been reported to exhibit antitumor activity (Cai et al., 2022; Gao et al., 2022). Ginsenoside Re modulates macrophage phenotype by interrupting the AMPKα1/stimulator of interferon genes (STING) positive feedback loop that promotes TAM M2 polarization and NSCLC migration; by suppressing this loop and the accompanying EMT, ginsenoside Re inhibits LLC tumor growth and metastasis in mouse models (Tang et al., 2024).

**Ginsenoside Rb1**, a representative metabolite of the *P. ginseng* C.A.Mey., *Panax notoginseng* (Burkill) F.H.Chen ex C. Chow, acts primarily on the cardiovascular system and cognitive function while also reversing multidrug resistance and enhancing chemotherapy efficacy (Ling et al., 2024; Shi et al., 2020; Jia et al., 2008). In PI3K-mutated NSCLC models, ginsenoside Rb1 inhibits tumor growth and metastasis, promotes M1 macrophage polarization, enhances CD8^+^ T cell proliferation and cytotoxicity, and remodels the immune microenvironment, with synergistic effects observed when combined with ICBs. These immune-modulating actions are mediated through inhibition of the PI3K/Akt/mTOR pathway and modulation of differentially expressed genes linked to drug resistance, including the programmed cell death PD-1/PD-L1 axis (Jin et al., 2025).

**Astragaloside IV**, a principal active constituent of *A. membranaceus* (Fisch.) Bunge, displays antioxidant, anti-inflammatory, anti-apoptotic, and immune-enhancing properties while inhibiting cancer cell migration and invasion and increasing chemosensitivity (Liang Y. et al., 2023; Zhang J. et al., 2020). In IL-4/IL-13/PMA-induced M2 macrophages derived from THP-1 cells, A549 and H1299 NSCLC cells, and Lewis lung carcinoma mouse models, astragaloside IV blocks macrophage M2 polarization by inhibiting the AMPK signaling pathway, as evidenced by downregulated CD206 and M2-associated genes, an effect partially counteracted by AMPKα silencing. *In vitro*, it suppresses lung cancer cell invasion, migration, and angiogenesis; *in vivo*, it inhibits tumor growth and metastasis while reducing the proportion of M2 macrophages (Xu F. et al., 2018).

These four glycosides, derived from Panax species and Astragalus membranaceus, converge on the reprogramming of TAMs through distinct molecular targets. Ginsenoside Rh2 and ginsenoside Re illustrate complementary strategies. Rh2 acts indirectly by suppressing tumor cell AURKA and glycolysis to reshape the immune milieu. Re disrupts an AMPKα1/STING feedback loop that sustains M2 polarization and EMT. Ginsenoside Rb1 adds a genotype-specific dimension by inhibiting PI3K/Akt/mTOR in PI3K-mutated NSCLC, concurrently promoting M1 polarization and CD8^+^ T cell activity. Astragaloside IV reinforces the theme of AMPK pathway blockade to suppress M2 polarization and angiogenesis. The amphiphilic triterpenoid scaffold common to these compounds enables membrane interaction and intracellular target engagement through the aglycone moiety, while the sugar chains modulate solubility and transport. This same structural complexity creates a consistent pharmacokinetic barrier across the class: the sugar moieties hinder intestinal absorption, and the aglycone undergoes rapid biliary excretion, resulting in poor oral bioavailability. Despite these limitations, their shared functional outcome is notable. Each compound attenuates the M2-oriented TAM niche and enhances antitumor immunity. Several demonstrate synergy with chemotherapy or immune checkpoint inhibitors.

### 3.6 Stilbenoids

Stilbenoids are phenolic compounds with a C6-C2-C6 skeleton and pentenyl substitution. They are found in edible plants and have attracted interest as potential dietary supplements because of their anti-inflammatory, antioxidant, and anticancer properties (Liu C. et al., 2026; Bo et al., 2023). The stilbene backbone consists of two aromatic rings linked by an ethylene bridge. This extended conjugation system gives them strong radical-scavenging and metal-chelating capacity. The multiple phenolic hydroxyl groups allow hydrogen bonding with protein targets and also contribute to membrane permeability. Methylated derivatives such as pterostilbene are more lipophilic and metabolically stable because they undergo less phase II conjugation, which gives them better oral bioavailability than the parent compound resveratrol.

**Resveratrol**, a stilbenoid widely distributed in grapes, berries, peanuts, and white quinoa, has well-established chemopreventive and anticancer activities (Rauf et al., 2018a; Ren et al., 2021). In Lewis lung carcinoma xenograft models and *in vitro* cultures of splenic granulocytic MDSCs (G-MDSCs) and monocytic MDSCs (M-MDSCs) from tumor-bearing mice, intragastric resveratrol reduces the splenic G-MDSC proportion and significantly lowers both total MDSCs and G-MDSCs within tumor tissue. After 12 h of *in vitro* stimulation, resveratrol promotes early and late apoptosis in G-MDSCs while upregulating CD11c and F4/80 surface expression on M-MDSCs, thereby driving their differentiation. Tumor growth is markedly suppressed, with clear reductions in both tumor volume and weight (Zhao Y. et al., 2019).

**3,4′,5-trimethoxystilbene**, an orally active resveratrol derivative, exhibits enhanced anticancer properties relative to the parent compound, including superior inhibition of proliferation, induction of cell cycle arrest, reduction of metastasis, and promotion of apoptosis (Aldawsari and Velázquez-Martínez, 2015). In PMA-induced THP-1 macrophages, H1299 and H1975 NSCLC cells, and subcutaneous NSCLC mouse models, trimethoxyresveratrol downregulates circular RNA PACRGL (circPACRGL) in both NSCLC cells and their exosomes, disrupting its interaction with insulin-like growth factor 2 mRNA binding protein 2 (IGF2BP2), reducing Yes-associated protein 1 (YAP1) mRNA stability, inhibiting Hippo signaling, and suppressing M2 macrophage polarization. *In vitro*, it blocks NSCLC proliferation, migration, invasion, and EMT; *in vivo*, it retards tumor growth and prevents exosome-mediated M2 polarization (Liu et al., 2024a).

**Pterostilbene** is a natural stilbenoid and a dimethylated analogue of resveratrol. It is found primarily in blueberries. Compared to resveratrol, it exhibits higher bioavailability and lipophilicity (Lin W. et al., 2020). The phenolic hydroxyl groups in its structure confer anti-inflammatory and antioxidant bioactivities. It has been investigated for anti-ageing, anti-obesity, and anticancer applications. (Ali M. et al., 2025). In A549 and H441 NSCLC cells and THP-1-derived M2-TAM models, pterostilbene simultaneously suppresses M2 polarization and lung cancer stem cell (LCSC) generation through the mucin 1 (MUC1)/NF-κB axis. Pretreatment of THP-1 cells with pterostilbene decreases the M2 proportion and increases the M1 proportion, while M2-TAM co-culture markedly expands the CD133^+^ stem cell subpopulation and tumor sphere formation capacity of A549 and H441 cells, effects that pterostilbene inhibits in a dose-dependent manner. (Huang W. et al., 2016).

These three stilbenoids share a resveratrol-derived scaffold yet achieve TME remodeling through mechanistically distinct routes. Resveratrol depletes G-MDSCs and drives M-MDSC differentiation. Trimethoxyresveratrol blocks an exosomal circRNA-protein axis to suppress M2 polarization. Pterostilbene simultaneously inhibits M2 polarization and LCSC generation *via* MUC1/NF-κB signaling. The C6-C2-C6 stilbene backbone confers potent radical-scavenging capacity through its extended conjugated system. The phenolic hydroxyl groups enable hydrogen bonding with protein targets and contribute to membrane permeability. Methylated derivatives such as pterostilbene exhibit enhanced lipophilicity and metabolic stability due to reduced phase II conjugation. This structural variation directly affects bioavailability and target engagement. The dimethylated analogue achieves superior oral exposure compared with the parent compound. These compounds demonstrate that stilbenoids can target MDSC populations, intercellular RNA communication, and the stem cell niche rather than relying on a single master pathway. The limited number of TAM-focused studies on this class precludes broad generalization. The favorable bioavailability of pterostilbene and the exosome-based mechanism of trimethoxyresveratrol, however, offer tractable leads for future investigation in NSCLC. Head-to-head comparisons of resveratrol and its methylated derivatives under identical experimental conditions would be particularly informative for understanding how methylation patterns affect TAM-modulating potency.

### 3.7 Lignans

Lignans are formed by the polymerization of two phenylpropanoid derivatives. They occur widely in nature and are structurally diverse; their basic parent nucleus consists of C6–C3 units linked at the β carbon. Several lignans have been used in the treatment of various malignancies, including non-small cell lung cancer (Wang J. et al., 2023; Zhong et al., 2025).

**Honokiol**, a natural phenolic compound extracted from the *Magnolia officinalis Rehder* and E.H.Wilson, exhibits therapeutic activity against cancer and other diseases (Ong et al., 2019; Rauf et al., 2018b). Formulations that enhance solubility and bioavailability increase its cytotoxicity toward NSCLC cells, and in mouse models honokiol reduces MDSC and M2 macrophage infiltration, inhibits the PI3K/Akt pathway to block M2 polarization, and synergizes with anti-programmed cell death protein 1 (anti-PD-1) therapy, leading to greater CD8^+^ T cell infiltration and activation (Cheng et al., 2024).

Honokiol currently provides the only TAM-related evidence available for lignans in NSCLC models. It combines M2 suppression, MDSC depletion, and anti-PD-1 synergy. These findings are encouraging but rest on a single compound. The high lipophilicity of the biphenyl scaffold probably aids cellular uptake, but with only one lignan evaluated under TME conditions, no structure–activity relationships can be inferred. The translational potential of lignans as TAM modulators in NSCLC will remain uncertain until a broader set of compounds is tested.

### 3.8 Phenolic acids

Phenolic acids carry a phenolic ring attached to a carboxyl group. They occur in botanical drugs such as rosemary and gallnuts and have been studied for roles in preventing diabetes, cardiovascular diseases, neurodegenerative disorders, and cancers (Ahmad et al., 2022; Arora et al., 2019). Their influence on TAM polarization likely involves targeting MAPKs and lowering oxidative stress, both of which are important drivers of M2 activation in NSCLC (Ahmad et al., 2022; Wan L. et al., 2019; Wang W. T. et al., 2024). The phenolic ring donates hydrogen atoms, giving these compounds their radical-scavenging ability, while the carboxyl group increases polarity and facilitates conjugation. As a result, they tend to have high aqueous solubility but are also susceptible to rapid renal clearance and poor gut stability (Li C. X. et al., 2023). Their relatively simple scaffold, however, offers a favorable safety profile and makes chemical modification for structural optimization straightforward.

**Rosmarinic acid**, a polyphenolic ester of caffeic acid and 3,4-dihydroxyphenyl lactic acid found in plants such as *Rosmarinus officinalis* L., *Rhus chinensis* Mill., possesses antiviral, antibacterial, anti-inflammatory, and antioxidant activities (Petersen and Simmonds, 2003; Zhao J. Y. et al., 2022). In a mouse peritoneal macrophage model that included control, rosmarinic acid, LPS-induced M1-polarized, and rosmarinic acid plus LPS treatment groups, a concentration of 20 μmol/L rosmarinic acid downregulated M1 markers, namely iNOS, TNF-α, and IL-1β, by inhibiting the expression of p-p65, a key component of the NF-κB pathway, while upregulating M2 markers such as Arg-1 and IL-10, improving LPS-induced M1 morphological features including reduced pseudopod formation, and enhancing macrophage viability (Huang Y. et al., 2025). In a NSCLC mouse model, rosmarinic acid treatment lowered tumor ROS levels by 60% and raised the M1/M2 ratio from 0.4 to 1.6; in HCC827 cell-MRC-5 fibroblast co-culture systems and xenograft models, it attenuated the expression of cancer-associated fibroblast (CAF) activation markers such as α-smooth muscle actin (α-SMA) and fibroblast activation protein (FAP), reversed cancer cell EMT, and, when combined with gefitinib, produced superior antitumor efficacy compared with monotherapy while delaying drug resistance (Li D. et al., 2025).

The TAM-focused evidence for phenolic acids in NSCLC is currently limited to a single compound, rosmarinic acid. This scarcity is itself informative. It highlights a significant research gap. Despite the well-established antioxidant and anti-inflammatory activities of this class, only one member has been evaluated for TAM modulation in NSCLC. The phenolic ring and carboxyl group combination confers favorable safety profiles and straightforward chemical modification potential. But the limited number of compounds evaluated in NSCLC prevents reliable structure-activity relationship inferences for this class. Additional phenolic acid compounds must be examined in NSCLC models before reliable mechanistic or structural patterns can be established.

Supplementary Table S2 summarizes the core mechanisms and application characteristics of the natural metabolites reported to modulate TAM polarization in NSCLC.

## 4 Multi-component botanical preparations

The preceding sections focused on purified single metabolites with well-defined structures and mechanisms that can be traced to specific molecular targets. A considerable body of evidence, however, comes from multi-component preparations. These include botanical extracts from defined plant sources and traditional Chinese medicine formulas that combine multiple botanical drugs. In such preparations, TAM polarization cannot be pinned on a single chemical entity, and the active constituents responsible for the observed effects are often only partially characterized. These preparations are therefore discussed separately below. It should be kept in mind that, without bioactivity-guided fractionation, mechanistic attribution remains inherently uncertain.

### 4.1 Botanical extracts

Botanical extracts are usually prepared from a single plant material by water or ethanol extraction. Their chemical composition is simpler than that of compound formulas, but their activity still reflects the combined contribution of multiple constituents. Several extracts have shown TAM-modulating effects *in vitro* and in tumor-bearing models. The available evidence is summarized below by source.

Marsdenia tenacissima extract (water extract) acts on hepatoma-derived growth factor (HDGF)-overexpressing PC-9 and H292 cells, THP-1-derived macrophages, H1975/PC-9 and M2 macrophage co-culture systems, and LLC C57BL/6 mouse models. The extract reduces HDGF expression and secretion in NSCLC cells, prevents M2 macrophage polarization, and inhibits NSCLC proliferation and migration. *In vivo*, it suppresses tumor progression, lowers plasma HDGF levels, decreases M2 infiltration, and raises the M1 ratio. Mechanistically, HDGF appears to promote IL-4 secretion by NSCLC cells, which then drives M2 polarization through the IL-4/JAK1/signal transducer and activator of STAT3 pathway; the extract interferes with this cascade by inhibiting HDGF and promoting M2-to-M1 repolarization (Fu J. et al., 2022).

*Scutellaria baicalensis* Georgi extract contains wogonin, baicalin, and baicalein (collectively termed WBB) as its major active constituents. It promotes M1 polarization and M2-to-M1 repolarization of macrophages through activation of the JAK2/STAT1 pathway, and this effect inhibits NSCLC tumor growth *in vivo*. Intratumoral CD8^+^ T cell infiltration remained low, however, and the increases in M1 macrophage infiltration and PD-L1 expression did not translate into enhanced efficacy of anti-PD-L1 therapy (Xiao Y. et al., 2021).

*Coptis chinensis* Franch. extract consists primarily of alkaloids, organic acids, coumarins, phenylpropanoids, and quinones (Wang J. et al., 2019). Recent work has focused on the flavonoid fraction of this extract, which includes baicalin. In LLC mouse xenograft models, leukotriene A4 hydrolase knockout (LTA4H^−^/^-^) mouse models, and *in vitro* systems, this fraction reduces M2-type TAM recruitment. It acts by blocking the leukotriene B4 (LTB4) signaling pathway through inhibition of LTA4H/BLT1. It also downregulates immunosuppressive factors such as IL-10 and maintains the proliferation and infiltrative capacity of CD8^+^ T cells. These effects inhibit tumor growth, enhance lung cancer sensitivity to ICBs such as PD-L1, and promote CTL-mediated tumor clearance in combination therapy (Yan et al., 2023).

*Gentiana macrophylla* flavonoids are extracted from *G. macrophylla Pall*., a traditional Tibetan botanical drug well recognized for its anti-inflammatory and antioxidant properties. Their major constituents likely include flavonoids such as isoorientin and vitexin (Mu et al., 2025). In the study reviewed here, the extract was prepared using 45% ethanol at 60 °C for 30 min with a solid-to-liquid ratio of 1:25, producing a flavonoid content of 22.13 mg/g. This extract inhibited NSCLC progression in A549 and H1299 cells and in nude mouse subcutaneous xenograft models. It acts through the circ_0059665/miR-512-5p/NOVA2 axis. Under hypoxic conditions, the extract downregulates circular RNA circ_0059665, which relieves its sponge effect on miR-512-5p and reduces downstream NOVA2 expression. As a result, NSCLC cell proliferation, invasion, and M2 macrophage polarization are suppressed. Overexpression of circ_0059665 or upregulation of NOVA2 reverses these antitumor effects (Ma et al., 2024).

### 4.2 Traditional Chinese medicine formulas

Compared with botanical extracts, traditional Chinese medicine formulas are more complex. They typically contain several to over a dozen botanical drugs, and the number of chemical constituents can reach tens or even hundreds. These formulas have shown antitumor activity in preclinical models, and some are already used as adjuvant treatments for advanced non-small cell lung cancer. The following sections introduce the experimental data formula by formula, focusing on their influence on TAM polarization and the signaling pathways involved. Because the link between active constituents and molecular targets remains largely unresolved in most formulas, the mechanistic interpretations offered here should be read with caution.

The Hugu Xiaoji Formula (HGXJ) inhibited primary NSCLC growth in mouse xenograft and bone metastasis models without notable toxicity. As monotherapy or in combination with immune checkpoint inhibitors (ICIs), it suppressed bone metastasis and secondary dissemination, reduced M2 macrophage polarization, enhanced CD8^+^ T-cell infiltration, and remodeled both the tumor and bone microenvironments. The proposed mechanism involves direct tumor suppression through the MAPK cascade and inhibition of M2 polarization *via* the signal transducer and activator of STAT3/6 pathway (Wang J. et al., 2025).

Xiaoji Decoction (XJD) acts on A549 and H1975 cells and mouse tumor models, exhibiting *in vitro* effects that inhibit NSCLC cell growth, promote apoptosis, induce cell cycle arrest, and downregulate the JAK1/p-STAT3/PD-L1 pathway. *In vivo*, it suppresses tumor growth, enhances the anticancer activity of αPD-L1, reduces M2 macrophages, and increases the proportion of M1 macrophages. Its mechanism involves inhibiting the JAK1/STAT3/PD-L1 pathway; combination therapy enhances caspase activation to promote apoptosis (He C. et al., 2025).

Bufei Decoction (BFD) acts upon PMA + IL-4-induced M2-polarized TAMs, A549/H1975 cells, and nude mouse xenograft models, blocking the tumor-promoting connection between TAMs and NSCLC cells by inhibiting IL-10 and PD-L1 expression. *In vitro*, it exhibits dose-dependent inhibition of TAM-induced NSCLC proliferation, migration, and invasion. *In vivo*, it suppresses tumor growth while reducing IL-10, PD-L1, and CD206 expression (Pang et al., 2017).

Yiqi Chutan Formula (YQCTF) acts on an NSCLC mouse model. Through network pharmacology and UPLC-MS/MS analysis, its aqueous extract inhibits lung cancer xenograft growth, modulates the tumor immune microenvironment (TIME), upregulates T cell proportion, promotes DC maturation, increases the TAM M1/M2 ratio, and reduces Treg and immunosuppressive neutrophils. Mechanism: Targets 60 interconnected genes, negatively modulates cancer-associated inflammation, and downregulates pro-tumor neutrophils *via* IL-6, HIF-1α, and other pathways (Le-Xin et al., 2024).

Wenxia Changbu Formula (WCF) acts in a co-culture model of macrophages and NSCLC cells, as well as in an rCCL18-induced nude mouse metastasis model, by antagonizing CCL18, mediating Src signaling, and attenuating TAM-induced EMT. *In vitro*, it downregulates macrophage CD163 expression, reduces CCL18 levels, and inhibits NSCLC growth, invasion, and EMT; *in vivo*, it suppresses rCCL18-induced metastasis (Bi et al., 2025).

Shiyiwei Shenqi Tablets (SST) act upon the Lewis lung cancer model and macrophage depletion experimental systems by inhibiting glutamine metabolism, downregulating NAT8L, and upregulating ASPA to suppress NAA production. This alleviates NAA’s inhibition of NF-κB activation, thereby reprogramming macrophages (promoting M1-like polarization and suppressing M2-like polarization). *In vivo*, it delays tumor progression, enhances CD8^+^ T-cell immunity, and reduces M2-driven angiogenesis and metastasis; *in vitro*, it modulates macrophage phenotype and function (Wang M. et al., 2025).

The Qingre Huoxue Formula (QRHXF) modulates the TME in a Lewis lung cancer subcutaneous tumor model by inhibiting TREM2 and the PI3K/Akt/STAT6 pathway. As a monotherapy, it inhibits tumor growth, reduces extracellular matrix deposition, angiogenesis, and metastasis, while downregulating M2 macrophage infiltration. When combined with anti-PD-1 therapy, it exhibits synergistic effects, enhancing the antitumor immune response (Li B. et al., 2025).

The Qiyu Sanlong Decoction (QYSLD) acts upon the C57BL/6 mouse LLC model and IL-4-induced M2 macrophage system by activating the PTEN pathway, thereby increasing the M1/M2 ratio in tumor tissues *in vivo* and modulating T cell subsets. *In vitro*, it reduces M2 markers and associated factors while increasing M1-related factors and activating CD4^+^/CD8^+^ T cells (Xu W. et al., 2025).

The Huoxue Huashi Decoction acts upon THP-1-induced M0 macrophages and NSCLC cell systems. Through network pharmacology screening, it targets TP53. By inhibiting TP53 expression, it upregulates macrophage CD86 and IL-1β whilst downregulating CD206 and CCL18, thereby inhibiting M2 polarization and significantly suppressing NSCLC invasion (Gao L. et al., 2023).

The Fei Sheng Jin formula acts upon RAW264.7 macrophage and A549 cell systems, upregulating the Notch1 signaling pathway (promoting Hes1 expression of Notch1, Jagged1, and Hes1), and inducing macrophage M1-type polarization (increasing CD86, iNOS, and IL-1β). The supernatant from treated macrophages significantly inhibits A549 cell proliferation. Hes1 expression), inducing M1-type macrophage polarization (increasing CD86, iNOS, and IL-1β). The supernatant from treated macrophages significantly inhibited A549 proliferation, migration, and invasion (Yang M. et al., 2023).

Jinfu’an Decoction (JFAD) acts on H1650 cells and lung cancer models, downregulating p120ctn and p120ctn S288 phosphorylation, mediating Kaiso upregulation, and inhibiting cell proliferation, migration, and metastasis in a dose- and time-dependent manner (Sun Z. et al., 2018). In macrophage systems and lung cancer models, targeting CTNNB1 (β-catenin) to downregulate its expression induces M2→M1 polarization, enhances the anticancer effects of cisplatin, and mitigates adverse reactions (Tang Y. et al., 2023). Furthermore, analysis of clinical samples revealed that advanced NSCLC patients exhibit elevated M2 macrophage proportions and reduced CD8^+^ T-cell infiltration. High CTNNB1 expression correlates with poor prognosis when CD8^+^ T-cell numbers are diminished. JFAD improves prognosis by downregulating CTNNB1 (Tang Y. 2024). These findings suggest that CTNNB1 may be a potential therapeutic target.

The Astragalus-Lotus Combination Formula administered to patients with stage IIIB or IV (in combination with AP/DP chemotherapy) significantly enhances quality of life and physical performance, ameliorates traditional Chinese medicine syndromes, and reduces tumor marker levels such as CEA and CYFRA21-1 by regulating immune function through qi-tonifying and constitutional strengthening. Its efficacy surpasses that of chemotherapy alone (Cai H. 2024).

Yupingfeng San (YPFS), a classical TCM formula traditionally used for enhancing immune function, has recently been investigated for its potential to modulate TAM polarization through an integrated single-cell transcriptomics and network pharmacology approach. scRNA-seq profiling of stage I NSCLC identified AHSA1 (activator of heat shock 90 kDa protein ATPase activity 1) as a key regulator of macrophage polarization, with high AHSA1 expression associated with poor prognosis. Network pharmacology analysis combined with molecular docking simulations subsequently revealed that several YPFS-derived bioactive flavonoids—including quercetin, kaempferol, and formononetin—exhibited strong predicted binding affinity to AHSA1, with quercetin showing the highest docking score. Based on these computational predictions, quercetin was inferred to possess the most potent inhibitory activity against M2 macrophage polarization among the tested metabolites. It must be emphasized that these findings are derived exclusively from computational analyses (network pharmacology and molecular docking) and currently lack experimental validation through *in vitro* or *in vivo* functional assays (Li H. et al., 2026).

Zi Shen Decoction (ZSD) is a classic traditional Chinese medicine compound. UHPLC-MS/MS analysis identified 92 compounds in ZSD. Network pharmacology predicted 233 core targets associated with TNF-α and T cell receptor signaling pathways. *In vitro*, ZSD showed no significant cytotoxicity on RAW264.7 cells. It effectively downregulated CD206 and Arg-1 (M2 markers) and upregulated CD86, iNOS, IFN-γ, and NO levels (M1 markers). M2 polarization was suppressed and M2→M1 reprogramming was induced. In LLC tumor-bearing mouse models, ZSD significantly inhibited tumor growth. It increased CD45^+^/F4/80^+^ macrophage infiltration in tumor tissues, elevated the CD86^+^ M1 proportion, and decreased the CD206^+^ M2 proportion. Serum IFN-γ and TNF-α levels were upregulated, while TGF-β2 levels were downregulated. 16S rRNA sequencing revealed that ZSD remolded gut microbiota composition and significantly increased the abundance of *Akkermansia*. An antibiotic (ABX)-induced pseudo-germ-free model validated the critical role of gut microbiota. After ABX-mediated microbiota depletion, the antitumor effects, macrophage polarization regulation, and cytokine modulation of ZSD were completely abolished. Notably, ABX treatment itself promoted M2 polarization, suggesting that microbiota dysbiosis drives M2 polarization (Sun H. et al., 2026).

Table 1 summarizes the effects and mechanisms of natural medicinal extracts or formulations in regulating macrophage polarization against NSCLC. These extracts or formulations exhibit distinct commonalities in NSCLC treatment-related research: They all target the TIME as a core pathway, focusing on inhibiting M2 macrophage polarization and activating M1 macrophages, enhancing CD8^+^ T cell infiltration, and regulating other immune subpopulations. This is achieved through mechanisms such as inhibiting classic oncogenic pathways like the STAT family, MAPK/JAK1/STAT3/PD-L1 pathways, and metabolic reprogramming. They consistently demonstrate tumor-suppressive activity *in vitro* and *in vivo*, exhibit synergistic effects when combined with immune checkpoint inhibitors or chemotherapeutic agents, and possess the advantage of low toxicity.

**TABLE 1:** Natural metabolite mixture regulates TAM polarization mechanism.

Name/abbreviation	Source/composition	Major active metabolites in traditional Chinese botanical drugs	Core polarization effect	Key signaling pathways	Main applications/properties	References
Marsdenia tenacissima extract (MTE)	*Marsdenia tenacissima* (Roxb.) Wight et al.	Kaempferol, quercetin, Apigenin, oleanolic acid, ursolic acid	Inhibits M2 polarization, promotes M2→M1 polarization, reduces M2 infiltration and increases M1 proportion	IL-4/JAK1/STAT3 (inhibits HDGF to interfere with this pathway)	Anti-NSCLC inhibits tumor proliferation, migration, and *in vivo* tumor progression	Fu J. et al., 2022
*Scutellariae Radix* extract (WBB)	*Scutellaria baicalensis* Georgi	Wogonin, baicalin, baicalein	Promotes M1 polarization and M2-to-M1 repolarization in a dose-dependent manner; increases proinflammatory factors; reduces anti-inflammatory factors	Activates JAK2/STAT1 pathway (upregulates JAK2, STAT1, and their phosphorylated forms)	Although WBB increased the infiltration of M1 macrophages in TME and upregulated the expression of PD-L1, WBB did not enhance the therapeutic effect of anti-PD-L1 in NSCLC.	Xiao Y. et al., 2021
*Coptis Chinensis* extract (RC)	*Coptis chinensis* Franch. (Contains alkaloids, flavonoids, *etc.*)	Berberine, Coptisine, Palmatine, jatrorrhizine, Magnoflorine	Reduces M2-type TAM recruitment, maintains CD8^+^T cell function	Inhibits LTA4H/BLT1 pathway	Anti-lung cancer, enhances ICBs (anti-PD-L1) sensitivity, promotes CTL-mediated tumor clearance in combination therapy.	Wang J. et al., 2019
*Gentiana macrophylla* flavonoids (GF)	*Gentiana macrophylla Pall*	Isorhamnoside, vitexin	Inhibit polarization of M2 macrophages	circ_0059665/miR-512-5p/NOVA2	Inhibit the proliferation and invasion of NSCLC cells; Inhibit tumor growth.	Ma et al., 2024
Hugu xiaoji Formula (HGXJ)	*Codonopsis pilosula* (Fisch.) Nannf., *Astragalus membranaceus* (Fisch.) Bunge, *Atractylodes macrocephala* Koidz., *Poria cocos* (Schw.) Wolf, *Angelica sinensis* (oliv.) Diels, *Ligusticum chuanxiong* Hort., *Paeonia lactiflora* Pall., *Rehmannia glutinosa* (Gaertn.) Libosch. ex Fisch. et Mey., *Lycium barbarum* L., *Cuscuta chinensis* Lam., *Eucommia ulmoides* oliv., *Drynaria roosii* Nakaike, *Hedyotis diffusa* Willd.	Astragaloside IV, Astragalus Polysaccharide, Calycosin, kaempferol, quercetin	Reduces M2 polarization, enhances CD8^+^T cell infiltration	MAPK cascade, STAT3/6	Anti-NSCLC and bone metastasis, effective alone or in combination with ICIs, remodels the tumor and bone microenvironment.	Wang J. et al., 2025
Xiaoji Decoction (XJD)	*Astragalus membranaceus* (Fisch.) Bunge, *Psoralea corylifolia* L., *Coriolus versicolor* (L.ex Fr.) Quél., *Hedyotis diffusa* Willd., *Buthus martensii* Karsch, *Scolopendra subspinipes* mutilans L. Koch, *Rheum palmatum* L.	Astragaloside IV, Astragalus Polysaccharide, Calycosin, kaempferol, quercetin	Reduces M2 macrophages, increases M1 proportion	JAK1/STAT3/PD-L1	Anti-NSCLC, induces apoptosis and cell cycle arrest *in vitro*, synergistic with αPD-L1	He, C., et al., 2025
Bufei Decoction (BFD)	*Codonopsis pilosula* (Fisch.) Nannf., *Schisandra chinensis* (Turcz.) Baill., *Rehmannia glutinosa* (Gaertn.) Libosch. ex Fisch. et Mey., *Astragalus membranaceus* (Fisch.) Bunge, *Aster tataricus* L. f., *Morus alba* L.	Codonopsis Polysaccharide, Lobetyolin, Atractylenolide III, Syringin, β-Sitosterol	Blocks protumor crosstalk between TAMs and NSCLC, downregulates M2 marker (CD206)	Inhibits IL-10 and PD-L1 expression	Anti-NSCLC dose-dependently inhibits tumor proliferation, migration, and invasion.	Pang et al., 2017
Yiqi Chutan Formula (YQCTF)	*Panax ginseng* C.A. Mey., *Arisaema erubescens* (wall.) Schott, *Fritillaria thunbergii* Miq., *Coix lacryma-jobi* L. var. *mayuen* (Roman.) Stapf, *Bufo bufo gargarizans* Cantor, *Gekko japonicus* Dumeril et Bibron, *Solanum nigrum* L., *Glehnia littoralis* Fr. Schmidt ex Miq.	Ginsenoside Rh2, ginsenoside Rg3, ginsenoside Re, ginsenoside Rb1, Protopanaxadiol	Increases TAM M1/M2 ratio	Targets 60 connecting genes, downregulates IL-6 and HIF-1α	Regulates tumor immune microenvironment, inhibits lung cancer xenograft growth	Le-Xin et al., 2024
Wenxia Changbu Formula (WCF)	*Aconitum carmichaelii* Debx., *Panax ginseng* C.A. Mey., *Angelica sinensis* (oliv.) Diels, *Rheum palmatum* L.	Ginsenoside Rh2, ginsenoside Rg3, ginsenoside Re, ginsenoside Rb1, Protopanaxadiol	Downregulates M2 marker (CD163), inhibits TAM-induced EMT	Src signaling (antagonizes CCL18)	Inhibits NSCLC growth, invasion, and metastasis (targeting rCCL18-induced metastasis)	Bi et al., 2025
Shiyi Weishenqi Tablet (SST)	*Panax ginseng* C.A. Mey., *Astragalus membranaceus* (Fisch.) Bge. Var. *mongholicus* (Bge.) Hsiao, *Asarum heterotropoides* Fr. Schmidt var. *mandshuricum* (Maxim.) Kitag., *Alisma orientalis* (sam.) juzep., *Cuscuta australis* R. Br., *Angelica sinensis* (oliv.) Diels, *Cassia obtusifolia* L., *Lycium barbarum* L., *Rehmannia glutinosa* (Gaertn.) DC., *Gastrodia elata* Bl., *Cervus nippon* Temminck	Ginsenoside Rh2, astragaloside IV, Astragalus Polysaccharide, Protopanaxadiol, Calycosin	Promotes M1 polarization and inhibits M2 polarization	NF-κB (alleviates NAA-induced inhibition of activation), glutamine metabolism pathway	Anti-Lewis lung cancer enhances CD8^+^T cell immunity, reduces angiogenesis, and metastasis	Wang M. et al., 2025
Qingre Huoxue Formula (QRHXF)	*Scutellaria baicalensis* Georgi, *Paeonia veitchii* Lynch	Baicalin, baicalein, Wogonoside, wogonin, luteolin	Reduces M2 macrophage infiltration	TREM2, PI3K/Akt/STAT6	Anti-Lewis lung cancer inhibits tumor growth alone, synergistic with anti-PD-1, and reduces ECM deposition and metastasis.	Li B. et al., 2025
Qiyu Sanlong Decoction (QYSLD)	*Astragalus membranaceus* (Fisch.) Bunge, *Polygonatum sibiricum* Delar. ex Redoute, *Gekko japonicus* Dumeril et Bibron, *Pheretima aspergillum* (E. Perrier), *Solanum nigrum* L., *Hedyotis diffusa* Willd., *Coix lacryma-jobi* L. var. *mayuen* (Roman.) Stapf, *Euphorbia helioscopia* L., *Curcuma zedoaria* (Christm.) Rosc., *Fritillaria cirrhosa* D. Don	Astragaloside IV, Astragalus Polysaccharide, Calycosin, kaempferol, quercetin	Increases M1/M2 ratio, reduces M2-related factors, increases M1-related factors	PTEN pathway	Anti-LLC lung cancer activates CD4^+^/CD8^+^T cells	Xu W. et al., 2025
Huoxue Huashi Decoction (HXHSD)	*Imperata cylindrica* (L.) Raeusch. Var. *major* (nees) C.E. Hubb., *Atractylodes macrocephala* Koidz., *Paeonia veitchii* Lynch, *Vigna angularis* (Willd.) ohwi et ohashi, *Rheum palmatum* L., *Codonopsis pilosula* (Fisch.) Nannf., *Poria cocos* (Schw.) Wolf, *Carthamus tinctorius* L., *Leonurus japonicus* Houtt., *Artemisia scoparia* Waldst. et Kit., *Zea mays* L., *Lycopus lucidus* Turcz. ex Benth., *Gardenia jasminoides* J.Ellis	Atractylenolide I, Atractylenolide III, Atractylon, Pachymic acid, β-Sitosterol	Inhibits M2 polarization, upregulates M1 markers (CD86, IL-1β)	Inhibits TP53 expression	Inhibits NSCLC invasion	Gao L. et al., 2023
Feijinsheng Formula (FJSF)	*Euphorbia helioscopia* L., *Cinnamomum cassia* Presl, *Panax ginseng* C.A. Mey., *Pinellia ternata* (Thunb.) Breit., *Salvia chinensis* Benth., *Cynanchum glaucescens* (Decne.) Hand.-Mazz., *Scutellaria baicalensis* Georgi, *Vespa affinis* (Linnaeus), *Arisaema erubescens* (wall.) Schott, *Zingiber officinale* Rosc., *Glycyrrhiza uralensis* Fisch.	Ginsenoside Rh2, ginsenoside Rg3, ginsenoside Re, ginsenoside Rb1, Protopanaxadiol	Induces M1 polarization (increases CD86, iNOS, IL-1β)	Notch1 (promotes expression of Notch1, Jagged1, Hes1)	Inhibits A549 proliferation, migration, and invasion	Yang M. et al., 2023
Jinfu’an Decoction (JFAD)	*Arisaema erubescens* (wall.) Schott, *Pinellia ternata* (Thunb.) Breit., *Fritillaria thunbergii* Miq., *Phragmites australis* (Cav.) Trin. ex Steud., *Coix lacryma-jobi* L. var. *mayuen* (Roman.) Stapf, *Pseudostellaria heterophylla* (Miq.) Pax ex Pax et Hoffm., *Gekko japonicus* Dumeril et Bibron, *Iphigenia indica* Kunth et Benth., *Salvia miltiorrhiza* Bunge, *Prunus persica* (L.) Batsch	Tanshinone IIA, cryptotanshinone, Danshensu, Salvianolic acid A, Salvianolic acid B	Induces M2→M1 polarization	Downregulates CTNNB1 (β-catenin), p120ctn	Anti-NSCLC enhances cisplatin efficacy, reduces adverse reactions, and improves the prognosis of advanced patients.	Sun Z. et al., 2018 Tang Y. 2024
Qilian mixture (QLM)	*Astragalus membranaceus* (Fisch.) Bunge, *Scutellaria barbata* D. Don, *Hedyotis diffusa* Willd., *Iphigenia indica* Kunth et Benth., *Coix lacryma-jobi* L. var. *mayuen* (Roman.) Stapf, *Prunella vulgaris* L., *Fritillaria thunbergii* Miq., *Glycyrrhiza uralensis* Fisch.	Astragaloside IV, Astragalus Polysaccharide, Calycosin, kaempferol, quercetin	Regulates immune function (no clear direct M1/M2 regulation, indirectly improves immune microenvironment)	Qi-invigorating and pathogen-eliminating related pathways	For stage IIIB/IV NSCLC, it improves quality of life and reduces tumor markers in combination with AP/DP chemotherapy	Cai, 2024
Yu Ping Feng San (YPFS)	*Saposhnikovia divaricate* (Turcz.) Schischk, *Astragalus membranaceus* (Fisch.) Bunge, *Atractylodes macrocephala* Koidz	Wogonin, kaempferol, quercetin	Computational prediction: Inhibits M2 polarization *via* AHSA1 binding	AHSA1 (predicted target, no experimental validation)	Predicted biological effects	Li, H. et al. 2026
Zi Shen Decoction (ZSD)	*Salvia miltiorrhiza Bunge and Glycyrrhiza uralensis Fisch*.	Flavonoids, phenolic acids, Diterpenes, Triterpene saponins, organic acids, and sugars.	M1 reprogramming (decreases CD206/Arg-1; increases CD86/iNOS/IFN-γ)	TNF/EGFR/Akt1	Inhibited tumor growth	Sun H. et al., 2026

## 5 Drug delivery: overcoming bioavailability barriers

Most natural metabolites suffer from poor bioavailability because of inherent characteristics such as low solubility, rapid intestinal metabolism, or swift systemic clearance, severely limiting their clinical translation and therapeutic efficacy. For example, curcumin undergoes extensive glucuronidation in the liver after oral administration, resulting in extremely low bioavailability (Noce et al., 2021); lentinan is readily degraded by the gut microbiota following oral intake, which reduces its bioavailability below therapeutic requirements (Gu et al., 2022); and most saponins have significantly restricted oral bioavailability owing to low intestinal absorption, poor membrane permeability, and rapid biliary excretion (Yu K. et al., 2012). Consequently, enhancing the bioavailability of natural metabolites has become a critical step for their translational application, and novel delivery systems such as nanocarriers, hydrogels, and exosomes have emerged as effective strategies to meet this challenge (Aljuffali et al., 2016; Fakhri et al., 2023; Fu et al., 2024; Hao et al., 2024; Zheng et al., 2024). Below, we discuss these delivery strategies according to the chemical class of the metabolite they are designed to deliver.

### 5.1 Flavonoids

Flavonoids such as baicalin and quercetin possess strong TAM-modulating activities but are limited by poor aqueous solubility and rapid metabolic clearance. Several delivery platforms have been developed to overcome these drawbacks while enabling macrophage-specific targeting.

Cerium oxide nanocomposites loaded with quercetin combine the intrinsic superoxide dismutase- and catalase-mimetic activities of nano-octahedral ceria with the antioxidant effects of quercetin. This combination effectively elevates the M2/M1 macrophage polarization ratio in inflammatory cell models, markedly downregulating pro-inflammatory cytokines and upregulating anti-inflammatory cytokines, thereby steering pro-inflammatory macrophages toward an anti-inflammatory phenotype and resolving inflammation (Wang Y. et al., 2021).

Hyaluronic acid (HA) nanoparticles exploit the overexpression of CD44 receptors on M2-type TAMs to achieve targeted delivery of baicalin and curcumin. These HA-baicalin/curcumin nanoparticles can selectively penetrate M2 pro-tumor macrophages and guide their polarization toward an M1 anti-tumor phenotype, exerting significant tumor-suppressive activity and modulating the TME (Berdiaki et al., 2021).

Biomimetic nanoparticle carriers further refine TAM-targeted delivery of baicalin. By inducing M2-type TAMs to polarize toward the M1 phenotype, they markedly enhance the infiltration of CD4^+^ and CD8^+^ T cells into tumor tissues, thereby activating anti-tumor immune responses. Both *in vitro* and *in vivo* studies confirm that this process effectively elicits CTL responses, directly inhibiting tumor growth while remodeling the immunosuppressive microenvironment (Han S. et al., 2019).

Poly (lactic-co-glycolic acid) (PLGA) nanoparticle carriers add environmental responsiveness: they react to the acidic lysosomal environment within the TME, enabling the precise release of baicalin. The antitumor effect involves a dual mechanism: direct tumor cell killing through the cytotoxic action of baicalin, combined with a synergistic interaction with the immunostimulant CpG. This drives M2-like TAMs toward M1-type polarization, prompting TAMs to secrete inflammatory cytokines that further reshape the immune microenvironment. *In vivo* and *in vitro* experiments have validated that this system can precisely target M2-like TAMs, and the TAM phenotypic reversal synergizes with the direct toxicity of baicalin to significantly enhance the overall anti-tumor effect (Han et al., 2021).

A nano-dandelion micelle system for the simultaneous delivery of curcumin and baicalin proved effective in overcoming tumor resistance, converting TAMs into tumor-killing M1-type macrophages and indicating potential as an adjuvant therapy (Ye et al., 2023).

### 5.2 Alkaloids

Alkaloids often display dose-dependent toxicity, exemplified by the hepatotoxicity induced by high-dose matrine, making controlled release and tumor-targeted delivery essential for improving their therapeutic index.

An *in situ* gel based on nano-graphene oxide (NGO) loaded with matrine (MAT-NGO-gel) addresses this issue through temperature-dependent drug release, thereby limiting systemic exposure. This system showed enhanced antitumor efficacy *in vivo*, particularly when combined with laser therapy, and was capable of inducing tumor cell apoptosis. An excellent safety profile with well-maintained body weight in mice was observed, providing an efficient, low-toxicity drug delivery system for the multi-mechanism treatment of malignant tumors (Chen et al., 2020).

Cell-penetrating peptide-modified liposomes have been employed to co-deliver the vinca alkaloid vinorelbine together with the steroidal saponin diosgenin. These liposomes enhance cellular uptake of both agents, display marked accumulation at tumor sites, and demonstrate favorable antitumor efficacy (Kong et al., 2020b). A related liposomal platform co-delivering RPV-loaded epirubicin and diosgenin further illustrates this principle by improving drug targeting to tumors, increasing selective accumulation at tumor sites, and exhibiting greater stability in the bloodstream (Kong et al., 2020a). These examples show that alkaloid delivery can be effectively combined with saponins to achieve synergistic targeting and stability.

### 5.3 Terpenoids

Terpenoids, particularly paclitaxel, are composed of isoprene units and are inherently lipophilic. This lipophilicity leads to limited aqueous solubility, recrystallization upon dilution, rapid blood clearance, non-specific tissue distribution, and co-solvent-induced toxicity; nano-delivery systems are therefore required to enhance water solubility and reduce these adverse effects (Ying et al., 2023).

Paclitaxel (PTX) is the most extensively studied terpenoid in this context. It is commonly formulated as human serum albumin-based nanoparticles encapsulating PTX, designated TENPA, which enable tumor-targeted accumulation (Chung H. et al., 2020). Alternatively, paclitaxel can be loaded onto exosomes derived from chimeric antigen receptor (CAR) T cells to generate PTX@CAR-Exos. These exosomes target mesothelin (MSLN)-expressing Lewis lung carcinoma cells through an anti-MSLN single-chain variable fragment (scFv) displayed on their surface, thereby overcoming the systemic toxicity associated with non-specific paclitaxel distribution (Zheng W. et al., 2023). Some researchers designed a folic acid (FA)-modified nano-herb micelle, in which PTX was first grafted with FA-linked polyethylene glycol (PEG) and then encapsulated with pristimerin (PRI) to form PRI@FA-PEG-PTX (P@FPP) nano-micelles (NMs). These NMs exhibited improved tumor-targeting capability and *in vivo* stability (Chen C. et al., 2022).

Artesunate, a water-soluble semisynthetic artemisinin derivative, still suffers from a short plasma half-life and non-specific tissue distribution. Artesunate-loaded PEGylated liposomes were prepared by the thin-film hydration method using a liposomal nanoformulation coated with DSPE-mPEG (2000). *In vitro*, this formulation exhibited markedly enhanced cytotoxicity against A549 cells, with IC_50_ values significantly lower than those of free artesunate. In a BALB/c mouse model of NSCLC, the PEGylated liposomes delayed tumor growth and prolonged overall survival compared with the control group (Qin Z. et al., 2026).

Tanshinone IIA (Tan IIA) is a lipophilic diterpenoid with potent anticancer activity, but its near-insolubility in water limits intravenous administration. One approach uses enzymatically hydrolyzed sodium caseinate (Eh-NaCas) as a natural nanocarrier: the hydrophobic compound loads into the protein’s hydrophobic domains through self-assembly, and the resulting Tan IIA-loaded Eh-NaCas nanoparticles (EhNaCas@TA) dramatically increase the apparent aqueous solubility of Tan IIA (Zhang J. et al., 2023). In a separate line of work, a biomimetic M1 macrophage membrane-coated nanoplatform, designated M1@CTP, has been employed for the co-delivery of Tan IIA and copper diethyldithiocarbamate (CuET). CuET- and Tan IIA-loaded PLGA nanoparticles (CTP) were first fabricated by a double-emulsion method and then coated with M1 macrophage membranes to yield M1@CTP. This platform achieves targeted co-delivery of both agents, converts immunologically “cold” tumors into “hot” tumors, elicits systemic antitumor immune responses, and shows a favorable safety profile (Bai X. et al., 2026).

Triptolide (TL) exhibits pronounced immunomodulatory and anticancer activities but is limited by severe systemic toxicity, including hepatotoxicity and reproductive toxicity. To address this, a study used TL and PTX to construct lipid-polymer hybrid nanoparticles (LPNs) as a co-delivery system (P/T-LPNs) *via* a nanoprecipitation method. The resulting formulation showed synergistic cytotoxicity against human lung cancer cell lines *in vitro* and significantly reduced tumor volume in a lung cancer-bearing mouse model, with low systemic toxicity (Liu J. et al., 2018).

### 5.4 Polysaccharides

Polysaccharides such as lentinan face unique delivery challenges because of their high molecular weight, hydrophilicity, and susceptibility to degradation by the gut microbiota. Polylactic acid nanoparticles loaded with lentinan (LNT@Mic) offer a tailored solution: they exhibit effective biocompatibility, allow controlled and sustained release of the polysaccharide payload, and reprogram the immunosuppressive TME. This strategy enhanced LNT bioavailability while improving the efficacy of anti-PD-L1 therapy in “cold” tumor models, with significant antitumor activity demonstrated across multiple tumor models including MC38 and 4T1 (Wang H. et al., 2023).

### 5.5 Saponins

Saponins suffer from poor membrane permeability and rapid biliary excretion, necessitating delivery systems that protect them from premature clearance while facilitating cellular uptake.

TMTP1-modified tumor-derived exosomes electroporation-loaded with ginsenoside Rb1 exemplify a dual-targeting strategy: the TMTP1 tumor-targeting peptide directs the carrier to tumor tissues, while the exosomal membrane shields the saponin payload from premature degradation, together enhancing tumor targeting as well as Rb1 stability and bioavailability (Jin X. et al., 2025).

A distinct but related approach employs plant-derived extracellular vesicle-like nanoparticles. Ginseng-derived nanoparticles (GDNPs), which naturally contain multiple ginsenosides and other bioactive constituents, promote M2-to-M1 macrophage polarization through the TLR4/myeloid differentiation primary response 88 (MyD88) signaling pathway. In B16F10 tumor-bearing mice, GDNPs significantly suppressed melanoma growth and increased M1 macrophage infiltration in tumor tissue (Cao et al., 2019). Unlike synthetic carriers loaded with a single purified metabolite, these naturally occurring nanoparticles deliver a cocktail of bioactive molecules, potentially mimicking the multi-component mechanism of the original plant material while achieving superior bioavailability.

### 5.6 Stilbenoids

Resveratrol is among the most intensively studied natural metabolites, yet its clinical utility is severely constrained by low aqueous solubility, poor chemical stability, limited bioavailability, and rapid *in vivo* metabolism. In cancer therapy, resveratrol has been encapsulated into a variety of nanocarriers, including liposomes, polymeric nanoparticles, lipid nanocarriers, and inorganic nanoparticles (Sarfraz et al., 2023). For instance, CD93-targeted PLGA nanoparticles loaded with resveratrol (RSV), designated CD93-NPs@RSV, have been shown to act on the CD93/Akt/PAK5/AIF signaling axis in Lewis lung carcinoma models, inhibiting tumor progression, synergizing with anti-programmed cell death PD-1 therapy, and prolonging survival (Jiang and Li, 2026). In a separate study, liposomes composed of lecithin, cholesterol, and resveratrol were prepared by the thin-film hydration method. In an orthotopic lung cancer mouse model, these liposomes suppressed TLR4/NLRP3 inflammasome-related genes, remodeled the tumor immune microenvironment, and inhibited lung cancer cell proliferation and migration while inducing apoptosis (Chen C. et al., 2025).

Pterostilbene already possesses superior bioavailability relative to resveratrol, yet nanoformulation strategies have been pursued to further boost its tumor accumulation and immunomodulatory activity. In one approach, paclitaxel (PTX) and pterostilbene (PTE) co-loaded nanoparticles (PTX-PTE NPs) were prepared by anti-solvent precipitation, with polyvinylpyrrolidone K30 (PVP-K30) dissolved in ultrapure water as the aqueous phase. The resulting PTX-PTE NPs not only markedly improved the aqueous solubility of both PTX and PTE but also enhanced the anti-A549/T activity of PTX and reversed multidrug resistance (MDR) by inhibiting the expression of P-glycoprotein (P-gp) and CDK1 in A549/T cells. (Liang X. et al., 2025).

### 5.7 Lignans

Honokiol (HK) is severely limited by poor aqueous solubility and rapid hepatic metabolism. To address these shortcomings, one strategy constructs glutathione (GSH)-responsive, biodegradable polyphosphazene-based nanoparticles (Nano-HK) by directly polymerizing HK, a disulfide-containing linker, and hexachlorocyclotriphosphazene (HCCP) through covalent bonds. The covalent linkage keeps Nano-HK stable in plasma; once inside tumor cells, the disulfide bonds are cleaved by elevated intracellular GSH, triggering site-specific HK release. In an A375 melanoma xenograft model, Nano-HK suppressed tumor growth more potently than free HK, induced G2/M arrest and apoptosis, and caused no obvious toxicity. This strategy has so far been validated only in A375 melanoma cells and still requires independent verification in NSCLC cell lines and *in vivo* models (Liao D. et al., 2025).

### 5.8 Phenolic acids

Phenolic acids such as rosmarinic acid are noted for their potent anti-inflammatory and antioxidant activities, but their clinical use is severely hampered by rapid hepatic metabolism and poor gut stability. Structurally related polyphenols such as curcumin face similar pharmacokinetic limitations; for example, curcumin undergoes extensive glucuronidation after oral administration, and lipid nanoparticle formulations have been developed to counteract this rapid metabolism and improve its bioavailability (Liu K. et al., 2022).

For local sustained delivery, a rosmarinic acid-loaded hydrogel offers distinct advantages by minimizing systemic exposure while maintaining therapeutic concentrations. This system induces M2 polarization of macrophages and inhibits pro-inflammatory cytokine secretion, combining low toxicity, high rosmarinic acid utilization, and controlled release (Xiong et al., 2025). The hydrogel platform is particularly well-suited to water-soluble phenolic acids such as rosmarinic acid, which otherwise suffer from rapid renal clearance and poor gut stability.

## 6 Discussion

The preceding sections present a substantial body of preclinical evidence. Eight chemical classes of natural metabolites have been evaluated for their capacity to modulate tumor-associated macrophage polarization in non-small cell lung carcinoma (Figure 1). The data are uneven. Some compounds have been validated in both *in vitro* systems and *in vivo* tumor models. Others rest on single-cell-line assays or computational predictions. The field is rich in mechanistic hypotheses but thin in clinical translation. What follows is an attempt to step back from the individual data points and ask what patterns hold across classes, where the evidence is weakest, and what it would take to move this field forward.

**FIGURE 1:**
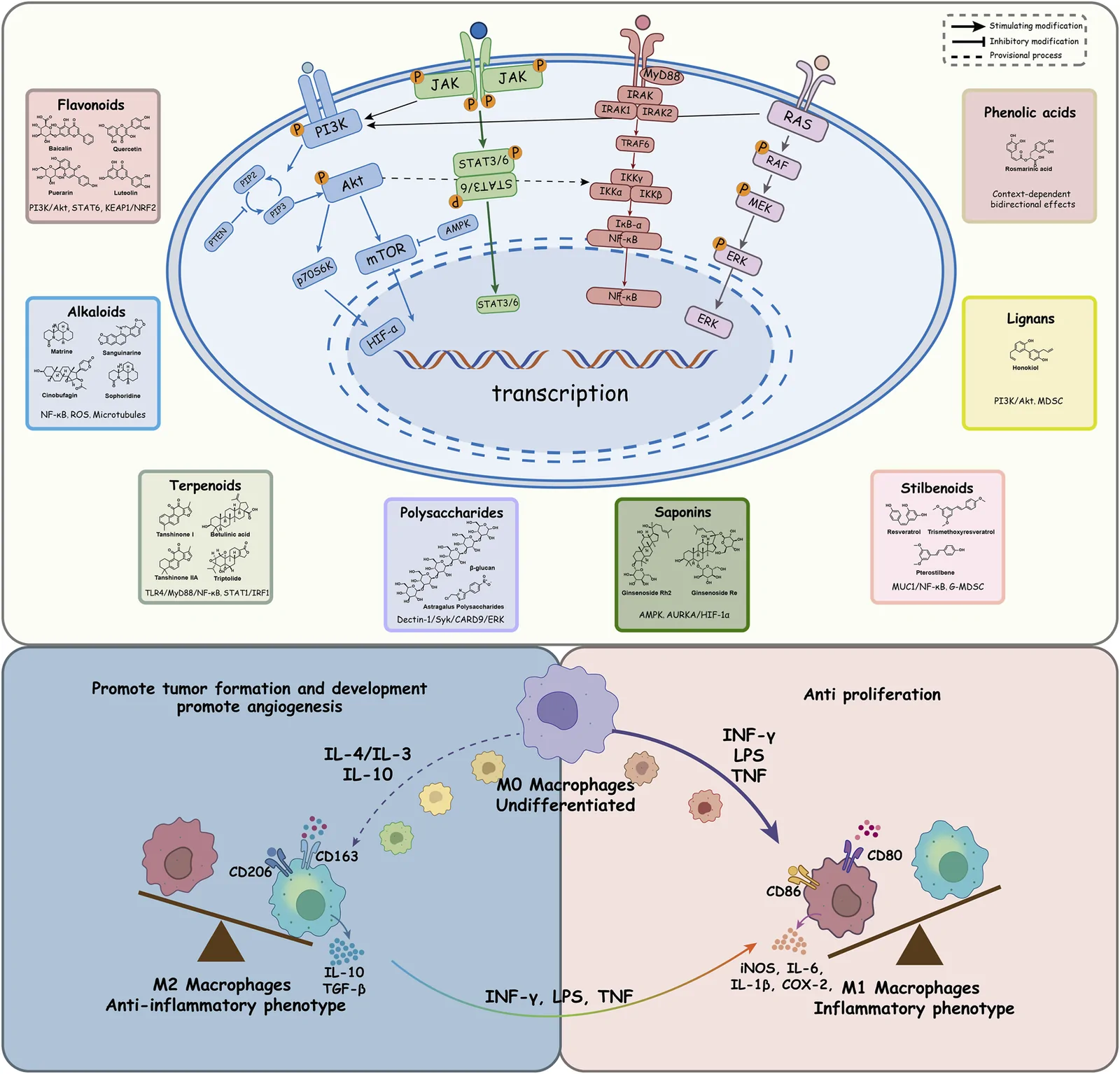
Schematic mapping of natural metabolite classes to their primary molecular targets in TAM polarization regulation for NSCLC.

### 6.1 Patterns across chemical classes

Macrophages are functionally plastic cells. In the TME, this plasticity is hijacked (Kloosterman and Akkari, 2023). Malignant cells remodel the surrounding immune landscape through intercellular interactions, and macrophages become drivers of cancer progression rather than guardians of tissue homeostasis. Reprogramming TAMs from a pro-tumor to an anti-tumor phenotype therefore has therapeutic potential (Cassetta and Pollard, 2018). The signals that drive macrophage polarization originate from cytokines, growth factors, and metabolites in the TME. They are transmitted through membrane receptors and downstream kinase cascades to the nucleus, where they reprogram gene expression and determine the polarization direction. Natural metabolites interfere with this process at various points along these signaling chains (Bai et al., 2025; Boutilier and Elsawa, 2021; Kerneur et al., 2022).

A few signaling pathways recur across structurally disparate compounds. PI3K/Akt/mTOR is the most frequently engaged node. Baicalin, matrine, betulinic acid, ginsenoside Rb1, and honokiol all converge on this axis, although they come from five different chemical families. NF-κB runs a close second. STAT family members appear as well, but the specific STAT varies with the compound: STAT6 for baicalin, STAT3 for 13-methylpalmatrubine, STAT1 for artesunate. This pattern may simply reflect the inherent constraints of macrophage biology, there are only so many pathways that control polarization, and any effective compound will eventually hit one of them. It may also suggest that our readouts are too coarse. Most studies measure a small set of surface markers and cytokines: CD206 and arginase-1 for M2, IL-1β and TNF-α for M1. A compound that upregulates CD86 and iNOS gets labeled “M1-promoting.” A compound that lowers CD206 and IL-10 gets labeled “M2-suppressing.” Whether these compounds achieve these effects through the same upstream events or through entirely different routes is rarely examined. The phenotypic convergence may obscure mechanistic divergence.

Beyond the shared pathways, each class shows some distinguishing tendencies. Flavonoids appear to couple redox modulation with immune effects. Baicalin engages KEAP1/NRF2/HO-1 in addition to its effects on STAT6 and PI3K/Akt. Whether this dual mechanism actually matters *in vivo* has not been tested. Alkaloids affect a broad range of targets, including microtubules, NF-κB, ROS, and exosome trafficking, and often combine direct tumor cell cytotoxicity with TAM-directed activity. The trade-off is toxicity. Matrine causes hepatotoxicity at high doses, and structural modifications aimed at reducing this have shown some promise, but the therapeutic window remains narrow. Terpenoids, particularly those that are highly lipophilic, show a striking tendency to synergize with immune checkpoint blockers. Paclitaxel, through TLR4-MyD88-NF-κB, and artesunate, through STAT1/IRF1, achieve similar endpoints, M1 repolarization and enhanced CD8^+^ T cell activity, *via* distinct mechanisms. The practical implication is that these compounds may be particularly well suited for combination regimens. Whether lipophilicity itself drives this synergy, perhaps through better penetration of the TME, or whether there is something more specific about the molecular targets, is not yet clear. Polysaccharides operate at the cell surface. They cannot cross membranes. Their effects on TAM polarization are mediated through pattern recognition receptors, including TLRs and Dectin-1. This is a fundamental difference from the small molecules reviewed in earlier sections. β-glucan engages Dectin-1/Syk/CARD9/ERK and triggers a metabolic shift toward glycolysis. This pathway is largely unique to this class. The downside is delivery: oral administration is inefficient, and nanoparticle encapsulation becomes necessary. Saponins cluster around AMPK signaling. Ginsenoside Re and astragaloside IV both target AMPK, though they come from different plants. Ginsenoside Rh2 works through AURKA/HIF-1α/glycolysis, and Rb1 through PI3K/Akt/mTOR. Whether the triterpenoid scaffold has some affinity for kinases in the AMPK family is an open question; no one has tested this systematically. Stilbenoids show diversity within a small family. Resveratrol depletes G-MDSCs. Trimethoxyresveratrol interferes with exosomal circRNA. Pterostilbene targets MUC1/NF-κB and suppresses cancer stemness. The methylation pattern of these compounds clearly affects bioavailability, but again, no systematic comparisons exist. Lignans and phenolic acids rest on the weakest evidence among the classes reviewed. Honokiol has been studied in NSCLC models and shows M2 suppression and MDSC depletion, but the mechanistic depth is limited, and no other lignan has been evaluated for TAM modulation in tumor systems. Rosmarinic acid promotes M2 polarization in acute inflammation, yet in the NSCLC microenvironment it shifts the M1/M2 ratio toward an anti-tumor profile. The same compound does opposite things depending on the setting, a reminder that macrophage polarization is a flexible state shaped by surrounding signals.

The multi-component botanical extracts and traditional Chinese medicine formulas described in Section 4 operate under a different logic. Their effects cannot be attributed to a single constituent. The active principles are often unknown. This makes mechanistic attribution inherently uncertain. Yet these preparations have shown consistent antitumor activity in preclinical models, and some have even entered clinical use as adjuvant therapies for advanced NSCLC. The Qilian Mixture, for example, has been evaluated in stage IIIB/IV patients receiving chemotherapy. It improved quality of life and reduced tumor marker levels. The Hugu Xiaoji Formula suppressed bone metastasis and enhanced CD8^+^ T cell infiltration. The Xiaoji Decoction increased the M1/M2 ratio and synergized with anti-PD-L1. These observations are clinically relevant but mechanistically opaque. The multi-component botanical extracts and traditional Chinese medicine formulas described in Section 4 operate under a different logic. Their effects cannot be attributed to a single constituent. The active principles are often unknown. This makes mechanistic attribution inherently uncertain. Yet these preparations have shown consistent antitumor activity in preclinical models, and some have even entered clinical use as adjuvant therapies for advanced NSCLC. The Qilian Mixture, for example, has been evaluated in stage IIIB/IV patients receiving chemotherapy. It improved quality of life and reduced tumor marker levels. The Hugu Xiaoji Formula suppressed bone metastasis and enhanced CD8^+^ T cell infiltration. The Xiaoji Decoction increased the M1/M2 ratio and synergized with anti-PD-L1. These observations are clinically relevant but mechanistically opaque.

What distinguishes the formulas from purified compounds is not just complexity but directionality. A single metabolite like baicalin hits a defined set of targets. A formula like ZSD introduces dozens, if not hundreds, of compounds into a biological system. Some of these components may act synergistically. Others may antagonize each other. The net effect, M1 repolarization, reduced M2 infiltration, and enhanced T cell activity, emerges from this aggregate activity. Whether this is more or less desirable than a clean single-target profile depends on the context. For drug development, specificity is valued. For clinical application, particularly in a disease as heterogeneous as NSCLC, a broader, more systemic effect may be advantageous.

ZSD provides an example. The formula’s TAM-modulating effect depended entirely on the presence of an intact gut microbiota. Antibiotic treatment abolished the antitumor activity and the polarization shift. This is a mechanism that no purified metabolite in this review has been shown to engage. It suggests that some formulas work indirectly, through the host microbiome, rather than through direct engagement of tumor or immune cells. This is both a strength and a complication. It opens a new avenue for understanding how botanical drugs work. It also makes the activity difficult to standardize, since the microbiome varies across individuals.

Combining natural metabolites with conventional therapies represents a promising strategy. Preclinical studies have suggested synergistic effects with chemotherapy and immune checkpoint blockers, and some formulations appear to mitigate treatment-related toxicities. These observations are preliminary, but they point toward a vision in which natural metabolites function not as standalone therapies but as adjuncts that enhance the efficacy of existing treatments while reducing their side effects (Dasari et al., 2022; Matsuo, 2023; Sheng et al., 2022; Yu B. et al., 2022).

### 6.2 Limitations

A few caveats are worth holding in mind. The first concerns the M1/M2 framework itself. These categories are useful heuristics, but they do not capture the range of functional states that macrophages adopt in tumors. TAMs possess significant phenotypic plasticity and can shift between polarization states, yet most drug studies still treat M1 and M2 as two isolated endpoints (Zhang W. et al., 2020). This binary view may overlook intermediate states that have distinct functional properties. Some TAMs express both M1 and M2 markers simultaneously. Others sit in states that fit neither category. A compound that shifts macrophages toward an intermediate state might be missed entirely if the readouts only look for full M1 or full M2 conversion.

The second caveat is contextual. Several compounds reviewed here show pro-M2 activity in acute inflammatory models. This is not an inconsistency. In acute inflammation, the problem is excessive M1-driven tissue damage, so shifting toward an M2 repair phenotype is protective. In the NSCLC microenvironment, the problem is persistent M2-driven immunosuppression, so the goal is to shift away from it. The same compound can do both, depending on the setting. This is not a flaw in the evidence; it is a property of the biology. But it does mean that studies using LPS stimulation or other acute inflammatory models should not be interpreted as evidence for or against efficacy in NSCLC. They are different contexts.

The third concerns model systems. PMA-differentiated THP-1 macrophages and IL-4/IL-13-induced RAW264.7 cells are reproducible platforms. They have enabled the discovery of many polarization-modulating compounds. But they are not the TME. The actual TAM phenotype is shaped by tumor-derived factors, hypoxia, metabolic gradients, and interactions with other immune cells (Cassetta and Pollard, 2023; Locati et al., 2020). A compound that shows strong M1 polarization in a simple two-cell system may not do the same in a tumor. This does not invalidate the *in vitro* findings, but it does mean that *in vivo* confirmation is essential. Some compounds have it; many do not.

The fourth caveat is technical. Some of the compounds reviewed here carry structural features that have been flagged as PAINS (Bolz et al., 2021). Polyphenols, quinones, and catechol-containing molecules can act as redox cyclers and metal chelators. They can generate false positives in cell-free assays and even in some cell-based systems. Vitexin and 13-methylpalmatrubine fall into this category. Their reported activity should be treated as preliminary until confirmed by orthogonal methods. This is not to say they are inactive, but the evidence is weaker than for compounds that have been validated in multiple models.

Finally, there is the question of structure-activity relationships. The field is not there yet. Almost all studies are single-compound, single-model investigations. Nobody has done systematic comparisons of structural analogues within a class under identical conditions. We cannot say with any confidence what part of the flavonoid scaffold is responsible for the redox-immune coupling, or which sugar chain on a ginsenoside is most important for AMPK engagement. These are not criticisms of individual studies; they are gaps in the collective knowledge that future work will need to address.

### 6.3 Clinical translation

Among the compounds reviewed here, only a handful have entered clinical trials. Vinblastine, paclitaxel, artesunate, and honokiol have all been studied in patients. In none of these trials was TAM polarization measured as an endpoint. The clinical benefits observed were attributed to direct cytotoxicity or general immunomodulation, not specifically to macrophage reprogramming. This is a significant gap. Baicalin, matrine, and ginsenoside Rh2 have extensive preclinical TAM polarization data, but none have entered trials for NSCLC. Lentinan has been evaluated in a meta-analysis of 1390 patients, but the endpoints were response rate and quality of life, not TAM phenotype.

The multi-component formulas have fared differently in this regard. Several are already in clinical use as adjuvants. The Qilian Mixture has been studied in a clinical setting with chemotherapy, showing improvements in performance status and reductions in tumor markers. This is not a trivial observation. It suggests that these preparations are tolerable and may offer some benefit. The problem is that we do not know whether the benefit comes from TAM repolarization, direct cytotoxicity, or some other effect. The endpoint data are too coarse to distinguish these possibilities. Also worth noting: most clinical studies of these formulas are not randomized, double-blind, controlled trials, and the sample sizes are small. The observed improvements cannot yet rule out placebo effects or selection bias, and the level of evidence remains limited.

The absence of TAM-focused clinical investigation is not surprising. These are early-stage compounds, and the field is still developing the tools to measure TAM polarization in patients. But the gap is notable. Until we have biopsy-based studies that assess CD163^+^/CD206^+^ and CD86^+^/iNOS^+^ before and after treatment, we will not know whether the TAM effects observed in mice actually occur in humans.

Biomarker development is a related priority. Baseline M2 macrophage density in tumor tissue is a reasonable candidate. Patients with high M2 infiltration might be the best candidates for M2-to-M1 reprogramming strategies. PD-L1 expression also deserves attention, given that several compounds interact with the PD-1/PD-L1 axis. Peripheral blood myeloid cell subsets could provide a minimally invasive surrogate, though their correlation with intratumoral TAM states is not well established (Cui et al., 2025).

Future trials should include paired biopsies. They should be designed around TAM endpoints, not just radiological response. They should stratify patients based on myeloid biomarkers. And they should test combinations with immune checkpoint blockers, because the preclinical synergy across multiple classes is too consistent to ignore. These are expensive, difficult studies. But without them, the field will remain where it is: rich in mechanism, poor in translation. The application of these compounds could also benefit from emerging computational tools. Molecular docking and machine learning approaches may accelerate the screening and optimization of natural product derivatives with improved TAM-targeting specificity (Chadokiya et al., 2024; Li Z. et al., 2022). These tools cannot replace biological validation, but they could help prioritize which analogues to synthesize and test, reducing the time and cost of hit-to-lead development.

Delivery remains a parallel challenge. The choice of carrier depends on the physicochemical nature of the metabolite: hydrophobic flavonoids, terpenoids, and some phenolic acids are best handled by lipid-based nanoparticles, PLGA carriers, or polymeric micelles, whereas water-soluble but metabolically fragile compounds such as rosmarinic acid are more suited to hydrogel-based local release. Hydrophilic polysaccharides need polymeric shields to avoid premature enzymatic breakdown, and saponins, which cross membranes poorly, benefit from exosomal or liposomal packaging. Targeting capability has improved from passive tumor accumulation *via* the enhanced permeability and retention effect to active ligands such as hyaluronic acid engaging CD44 on M2 macrophages (Mattheolabakis et al., 2015), folic acid for tumor-directed delivery (Sharma et al., 2026), TMTP1 peptide-guided exosomes, and CD93-targeted nanoparticles. Environment-responsive carriers that release cargo in acidic lysosomal conditions or under elevated intracellular glutathione help confine drug release to the TME. Biomimetic platforms, including M1 macrophage membrane-coated nanoparticles and ginseng-derived extracellular vesicle-like nanoparticles that promote M2-to-M1 polarization through TLR4/MyD88 signaling, add immunomodulatory functions beyond those of the payload alone. Co-delivery and combination strategies, such as paclitaxel co-loaded with triptolide or curcumin co-encapsulated with baicalin, have produced greater antitumor activity than single agents, and several nanocarrier systems have been tested together with immune checkpoint inhibitors. Still, obstacles remain. Improvements in bioavailability are rarely quantified in a standardized manner; some immunological validations were performed in inflammatory rather than tumor-bearing models; targeting ligands like CD44-binding hyaluronic acid and TMTP1 peptide are not uniquely expressed on M2 TAMs, raising the possibility of off-target effects; and inorganic carriers such as nano-graphene oxide lack thorough long-term safety and degradation data. A tighter match between carrier design and metabolite properties, combined with standardized pharmacokinetic reporting and rigorous toxicological evaluation, will be necessary to advance these delivery strategies toward TAM-directed applications in NSCLC.

## 7 Conclusion

The convergence of *in vivo* efficacy across eight chemical classes provides a strong preclinical signal. Natural metabolites can repolarize TAMs through multiple routes, and several have already shown synergy with immune checkpoint blockers in animal models. But the field is caught in a familiar trap: mechanistic richness without clinical translation. The tools to measure TAM polarization in patients exist. Biopsy-based trials with TAM endpoints are feasible. The missing element is not technology but design. Until clinical studies ask whether macrophage repolarization actually occurs in humans, the translational promise of these compounds will remain a hypothesis. The preclinical evidence is no longer the bottleneck. The clinical question is.
