# Supplementary material for: Natural metabolites targeting macrophage polarization for non-small cell lung cancer therapy: a review of mechanisms and applications based on chemical classification
Source: Front Pharmacol. 2026 Sep 25;17:1856152. doi: 10.3389/fphar.2026.1856152 (PMC13650678; doi:10.3389/fphar.2026.1856152)
Supplement: Supplementary file 1 [file Supplementaryfile1.pdf]

1 Literature Search and Study Selection

1.1 Search Strategy

A systematic literature search was performed across three authoritative biomedical databases: PubMed, Cochrane Library, and Google Scholar. The search covered all records from database inception to July 1, 2026.

The search strategy was constructed based on three core dimensions: disease, target, and intervention, combined with Boolean operators.

For English databases (taking PubMed as a representative example), the full search string was as follows:

#1 ("Carcinoma, Non-Small-Cell Lung" OR "non-small cell lung cancer" OR NSCLC)

#2 ("Tumor-Associated Macrophages" OR "tumor-associated macrophage" OR TAM OR "macrophage polarization" OR "M1/M2 polarization")

#3 ("Natural Products" OR "natural metabolites" OR "natural compounds" OR flavonoid* OR alkaloid*OR terpenoid* OR "phenolic acid" OR polysaccharide*OR saponin*OR lignan*OR "traditional Chinese medicine")

#4 #1 AND #2 AND #3

Minor syntax adjustments were applied to each database to match their respective retrieval rules, while the core search logic remained consistent across all platforms.

1.2 Inclusion and Exclusion Criteria

Inclusion criteria

Studies were eligible for inclusion if they met all of the following criteria:

1. Study type: Original experimental research, including in vitro cell-based assays, in vivo animal tumor models, and clinical studies;

2. Research subject: Studies performed in non-small cell lung cancer-related models (cell lines, tumor-bearing animals, or clinical patients);

3. Intervention: Natural monomers, plant extracts, or traditional Chinese medicine formulas with explicitly reported regulatory effects on TAM polarization;

4. Outcome measures: Studies reporting data on M1/M2 polarization markers, associated signaling pathways, tumor growth, or metastasis.

Exclusion criteria

Studies were excluded if they satisfied any of the following:

1. Non-original research, including review articles, editorials, conference abstracts, case reports, and patents;

2. Studies only investigating direct cytotoxicity on cells without addressing macrophage polarization regulation;

3. Studies conducted in disease models other than NSCLC;

4. Studies with unavailable full text, severely incomplete data, or duplicate publications.

The same inclusion and exclusion criteria were applied to ensure uniform evaluation standards.

1.3 Study Screening Process

Two independent researchers performed the literature screening in duplicate. Discrepancies were resolved through discussion with a third senior researcher to minimize subjective bias. The screening workflow is summarized as follows:

1. A total of 1339 records were initially retrieved from the three databases. After duplicate removal across all databases, 1077 unique records remained.

2. After title and abstract screening, 624 irrelevant records were excluded, and 453 articles were retained for full-text assessment.

3. After full-text evaluation against the inclusion and exclusion criteria, 389 articles were excluded (168 lacked mechanistic data on TAM polarization, 109 used non-NSCLC models, 112 had incomplete or uninterpretable data).

4. Ultimately, 64 original research articles were included in this review.

A PRISMA flow diagram has been added as Supplementary Figure S1 to visually present the complete literature screening process.

1.4 Quality Assessment and Evidence Level Classification

All metabolites included in this review were assessed using the PAINS filters (Bolz et al., 2021), and compounds bearing known PAINS structural alerts (e.g., catechol-containing flavonoids and quinone-bearing compounds) are explicitly annotated. In addition, an evidence-level classification system was applied to each metabolite reviewed: Level A (Clinical evidence for TAM polarization modulation), Level B (In vivo + in vitro TAM-specific evidence), Level C (In vitro only), and Level D (Indirect evidence). The detailed evidence classification table is provided in Supplementary Table S3.

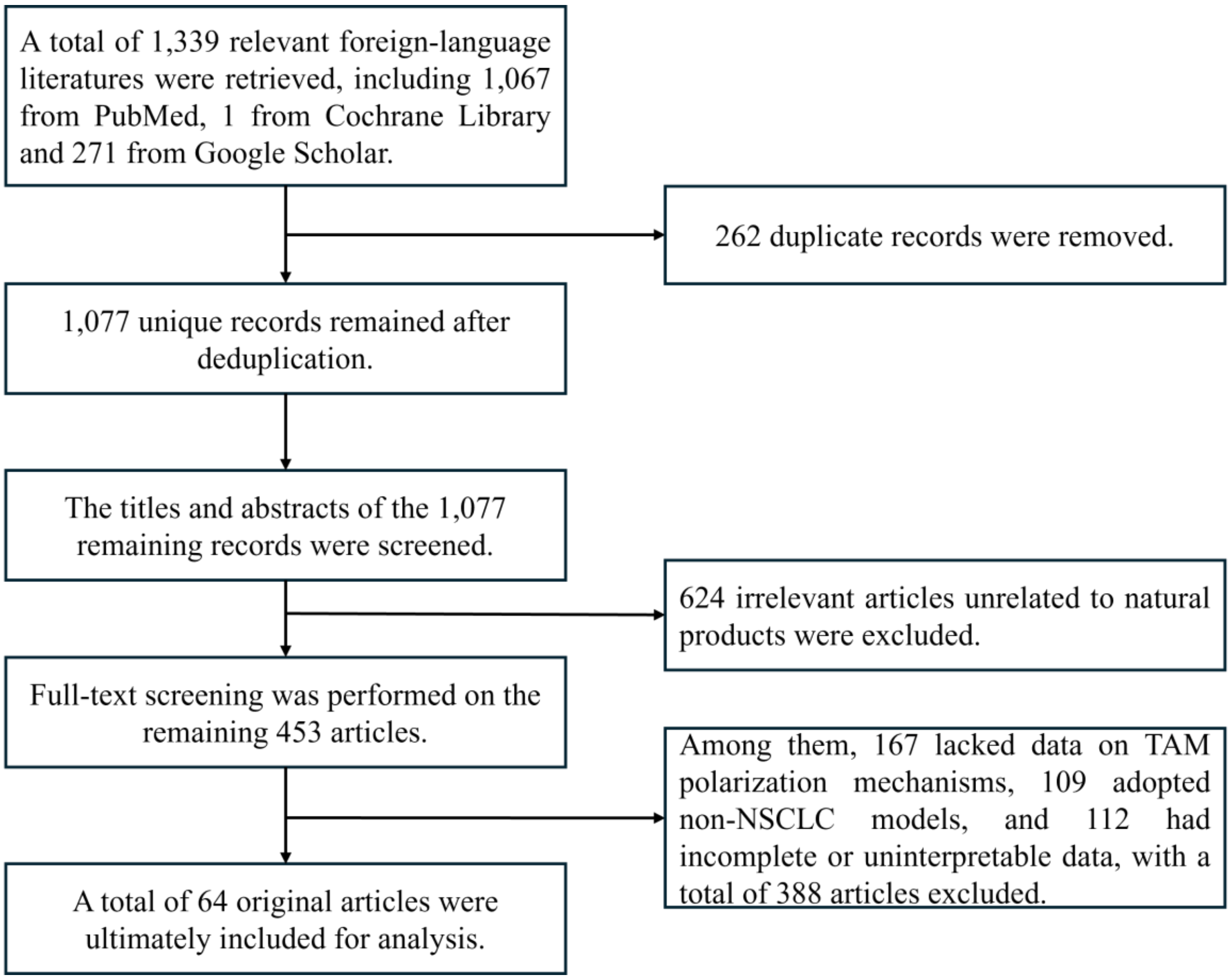

Figure S1 PRISMA flow diagram
