# Supplementary material for: Natural metabolites targeting macrophage polarization for non-small cell lung cancer therapy: a review of mechanisms and applications based on chemical classification
Source: Front Pharmacol. 2026 Sep 25;17:1856152. doi: 10.3389/fphar.2026.1856152 (PMC13650678; doi:10.3389/fphar.2026.1856152)
Supplement: Supplementary file 2 [file Table1.pdf]

Table S1 Evidence Level Classification

**Chemical Class**	**Metabolite**	**Evidence Level**
**Flavonoids**	Baicalin	B
	Puerarin	D
	Quercetin	C
	Luteolin	D
	Kaempferol	C
	Formononetin	D
	Vitexin	D
	Isoginkgetin	B
**Alkaloids**	Vinblastine	D
	Matrine	B
	Sophoridine	B
	Sanguinarine	B
	Cinobufagin	C
	13-Methyl-palmatrubine	B
**Terpenoids**	Paclitaxel	D
	Tanshinone I	B

Chemical Class	Metabolite	Evidence Level
	Tanshinone IIA	B
	Dihydrotanshinone I	B
	Artesunate	B
	Betulinic acid	B
	Triptolide	B
	β-Elemene	B
	Scutebarbatine A	B
	Tubeimoside II	B
**Polysaccharides**	Astragalus Polysaccharides	B
	β-Glucan	B
	Lentinan	C
**Saponins**	Ginsenoside Rh2	B
	Ginsenoside Re	B
	Ginsenoside Rb1	B
	Astragaloside IV	B
**Lignans**	Honokiol	B

**Chemical Class**	**Metabolite**	**Evidence Level**
**Phenolic acids**	Rosmarinic acid	B
**Stilbenoids**	Resveratrol	D
	Trismethoxyresveratrol	D
	Pterostilbene	C

Note: Level A (Clinical evidence for TAM polarization modulation), Level B (In vivo + in vitro TAM-specific evidence), Level C (In vitro only), and Level D (Indirect evidence).
