# Supplementary material for: Natural metabolites targeting macrophage polarization for non-small cell lung cancer therapy: a review of mechanisms and applications based on chemical classification
Source: Front Pharmacol. 2026 Sep 25;17:1856152. doi: 10.3389/fphar.2026.1856152 (PMC13650678; doi:10.3389/fphar.2026.1856152)
Supplement: Supplementary file 3 [file Table2.pdf]

Table S2: The mechanism of natural metabolite monomers regulating TAM polarization

Category	Natural metabolite	Chemical structure	Source	Animal/Cell Models	**Dose**	Key points of Action	**References**
Flavonoids	Baicalin*	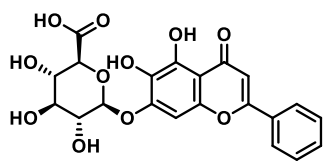	*Scutellaria baicalensis* Georgi	(i) *In vitro* RAW264.7 murine macrophages; Hela cells; LPS stimulation;<br>(ii) *In vitro* A549; A549/DDP; RAW264.7;<br>*In vivo* A549 or A549/DDP subcutaneous tumor-bearing mouse model;<br>(iii) *In vitro* Human A549 lung cancer cells; Cisplatin-resistant human A549/DDP lung cancer cells; Murine RAW264.7 macrophage cells;<br>*In vivo* A549 lung cancer-bearing mice (ectopic xenograft model);	(i) *In vitro* 0.01, 0.1, 1, 10 μM;<br>(ii) *In vitro* 8 μg/mL;<br>*In vivo* 100 mg/kg, i.g., qd× 21 d<br>(iii) *In vitro* 0.1, 1, 10 mM;<br>*In vivo* 1 mg/g, i.v., q2d × 20 d	⊣ PI3K/Akt/mTOR, STAT6; → KEAP1-NRF2/HO-1	(i) Fan, G., et al., 2013;<br>(ii) Chen, Y., et al., 2024;<br>(iii) Wang, B., et al., 2019;
	Puerarin	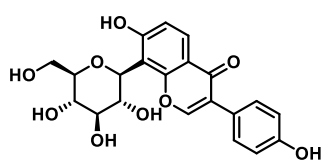	*Pueraria montana* (Lour.) Merr.	*In vivo* Subcutaneous xenograft model of human A549 non-small cell lung cancer in NOD/SCID mice	*In vivo* 40 mg/kg, i.p., q2d × 30 d	⊣ MEK/ERK1/2	Kang et al., 2017
	Quercetin*	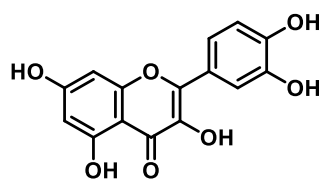	Widely present in fruits and vegetables	(i) *In vitro* RAW264.7 murine macrophages; IMG adult mouse microglia cell line;<br>(ii) *In vitro* PMA-differentiated THP-1 macrophages; IL-4/IL-13-induced M2 polarization; A549 cells; plasmid-transfected model systems	(i) *In vitro* 0, 5, 10 μM<br>(ii) *In vitro* 60 μmol/L	⊣ SPP1, AMPK/Akt (context-dependent)	(i) Tsai et al., 2021;<br>(ii) Wu & He, 2025
	Luteolin*	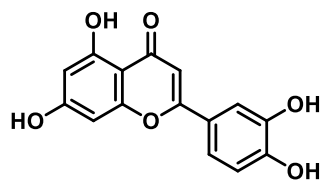	Vegetables, medicinal plants, fruits	*In vitro* A549 and H460 NSCLC cell lines	*In vitro* 20, 40, 80 μM	⊣ TAM RTK/Axl	Lee, Y., et al., 2017
	Kaempferol*	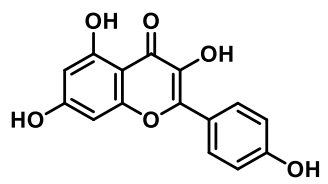	Flowers, cabbage, broccoli	*In vitro* PMA-differentiated THP-1 macrophages; IL-4/IL-13-induced M2 polarization	*In vitro* 50 μM	⊣ STAT1-associated macrophage genes	Wan, L., et al., 2025
	Formononetin	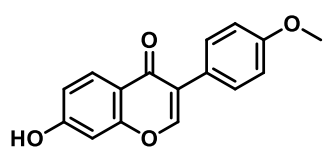	*Astragalus membranaceus* (Fisch.) Bunge	*In vivo* Lewis lung carcinoma mouse models	*In vivo* 25, 50, 100 mg/kg, i.g., qd ×10 d	⊣ AMPK, Wnt	Cai, H., 2024

	Vitexin	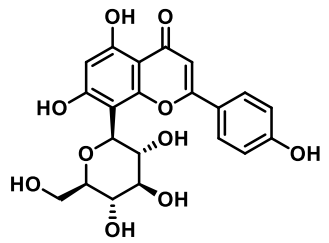	*Crataegus spp., Passiflora spp.*	*In vivo* M1/M2 polarization of murine RAW264.7 macrophages induced by LPS and IL-4	*In vivo* 25, 50, 100 μmol/L	⊣ p-STAT3	Zhao, B., et al., 2020
	Isoginkgetin	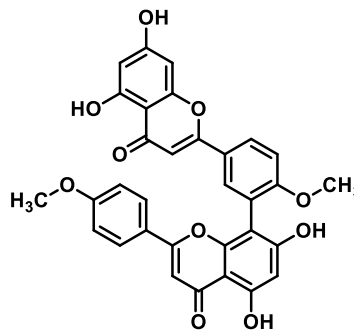	*Ginkgo biloba* leaves	*In vitro* BEAS-2B, THP-1, A549, H1299; *In vivo:* subcutaneous A549 xenograft model	*In vitro* 0, 40, 60, 80 μM; *In vivo* 30mg/kg, i.p., q2d, ×16 d	⊣ TRIB3 → Nrf2	Zhu et al., 2026
Alkaloids	Vinblastine	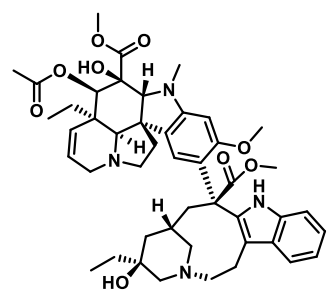	*Catharanthus roseus* (L.) G. Don	*In vivo* subcutaneous MC38 and LLC syngeneic tumor models	*In vivo* 1.25 mg/kg, s.c., q2d, ×12 d	→ NF-κB, CYBA-dependent ROS	Wang, Y., et al., 2023
	Matrine	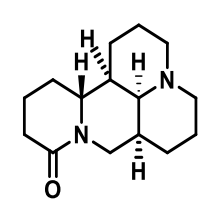	*Sophora flavescens* Aiton, *Radix Sophorae tonkinensis*	*In vitro* RAW264.7 M2 macrophage model induced by IL-13; *In vivo* LLC subcutaneous tumor model; LLC intravenous lung metastasis model	*In vitro* 20 μmol/L; *In vivo* 40, 80 mg/kg, i.g., qd × 16 d	⊣ mTOR/PI3K/Akt	Zhao, B., et al., 2021
	Sophoridine	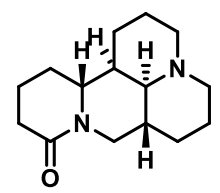	*Sophora flavescens* Aiton, *Radix Sophorae tonkinensis*	*In vitro* RAW264.7 and THP-1-derived macrophages; H460 and Lewis lung carcinoma cells; *In vivo* Lewis lung carcinoma mouse xenograft model	*In vitro* 20, 40 μg/ml; *In vivo* 15, 25 mg/kg i.p.25 d	→ NLRP3/caspase-1/GSDMD; ⊣ PI3K/Akt/mTOR	Luo et al., 2023
	Sanguinarine	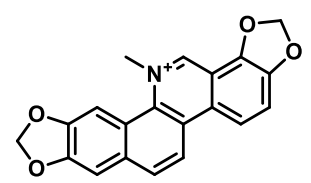	*Sanguinaria canadensis* L.	(i) *In vitro* Exosome-mediated co-culture model of A549 and THP-1 cells; (ii) *In vivo* LLC subcutaneous tumor-bearing mouse model	(i) *In vitro* 2, 4, 6, 8, 10 μM; (ii) *In vivo* 5 mg/kg, i.p., q2d × 20 d	→ MAPKs	(i) Yu, Y., et al., 2020; (ii) Li, B., et al., 2022
	Cinobufagin	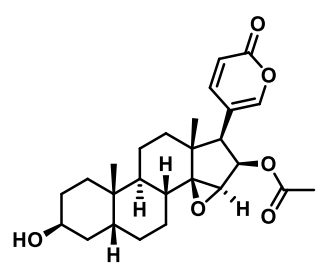	*Bufo gargarizans* Cantor	*In vitro* PMA-differentiated THP-1 macrophages; IL-4/IL-13-induced M2 polarization	*In vitro* 25 ng/ml	⊣ NF-κB (exosome-mediated); → MDSC apoptosis	Sun, Y., et al., 2025
	13-Methyl-palmatrubine	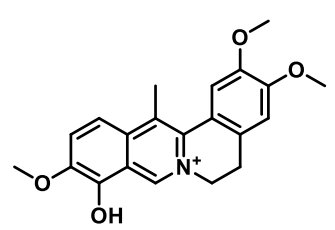	*Corydalis yanhusuo* W.T.Wang ex Z.Y.Su & C.Y.Wu	(i) *In vitro* A549 cell lines; *In vivo* A549 tumor-bearing nude mouse intraperitoneal transplantation model; (ii) *In vitro* A549, NCL-H1975; PMA-differentiated THP-1 macrophages; IL-4/IL-13-induced M2 polarization;	(i) *In vitro* 30, 60, 90 μg/ml; *In vivo* 3, 6, 9 mg/kg, i.p. q3d ×7; (ii) *In vitro* 30, 60, 90 μg/mL;	⊣ HIF-1α glycolytic reprogramming	(i) Chen, J., et al., 2016; (ii) Wu, Z., et al., 2022

				In vivo A549/NCL-H1975 subcutaneous xenograft model	*In vivo* 3, 9 mg/kg, i.p., qd × 28 d		
Terpenoids	Paclitaxel	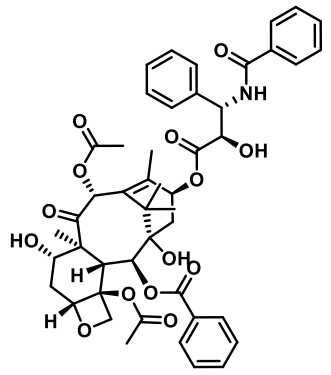	*Taxus* spp. (e.g., *Taxus chinensis* (Pilg.) Rehd.)	-	-	→ TLR4, type I/II interferon pathways	Kalinski et al., 2025
	Tanshinone I*	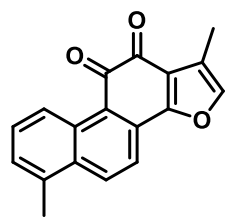	*Salvia miltiorrhiza* Bunge	*In vitro* PMA-differentiated THP-1 macrophages; IL-4/IL-13-induced M2 polarization; *In vivo* CL1-5 subcutaneous xenograft lung metastasis model in SCID mice	*In vitro* 1, 10, 20, 40, 80, 160 μg/mL; *In vivo* 0.3 mg/kg, i.p., qd× 30d	⊣ AP-1, NF-κB, Ras-MAPK, Rac1	Lee, C., et al., 2008
	Tanshinone IIA*	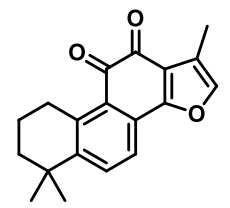	*Salvia miltiorrhiza* Bunge	(i) *In vitro* LPS-treated RAW264.7 macrophages, (ii) *In vivo* LPS-induced acute lung injury murine model; (iii) *In vivo* H1975 subcutaneous xenograft model; LLC subcutaneous tumor model; LLC-bearing C57 mouse model for Tan IIA combined anti-PD-1 immunotherapy	(i) *In vitro* 0.1, 1, 10 Mm; (ii) *In vivo* 10 mg/kg, i.p. qd ×3d (iii) *In vivo* 10, 20, 40 mg/kg, i.p., qd×18d; 15, 30, 60 mg/kg, i.p., qd×18d; 40 mg/kg, i.p., qd×15d	⊣ miR-155; → TLR4-HMGB1/CEBP-β; ⊣ PD-L1	(i) Gao, S., et al., 2019 (ii) Zhao, J., et al., 2022 (iii) Zhang, Y., et al., 2024
	Dihydrotanshinone I	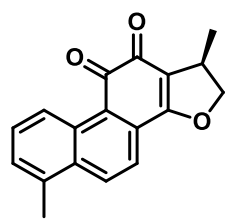	*Salvia miltiorrhiza* Bunge	*In vitro* LLC, A549 cell lines; *In vivo* LLC subcutaneous syngeneic cell transplantation tumor model; A549 subcutaneous xenograft cell transplantation tumor model	*In vitro* 4 μM; *In vivo* 20, 40 mg/kg, i.p. q3d ×4 time	⊣ PHGDH → ↓ Serine biosynthesis → ↓ SAM generation → ↓ H3K4me3 → ↓ IL-10/TGF-β transcription → Remodelled macrophage polarization → ↑ $CD8^+$ T cell-mediated	Qian, C., et al., 2026

						antitumor immunity	
	Artesunate	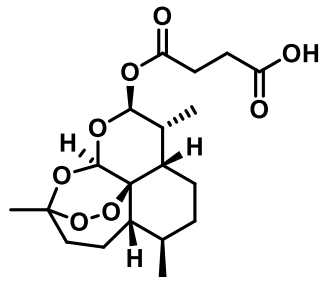	*Artemisia annua* L.	*In vitro* LLC, RAW264.7 cell lines;<br>*In vivo* LLC subcutaneous Lewis lung carcinoma xenograft model	*In vitro* 1, 5 ,10 μM;<br>*In vivo* 20 mg/kg, i.p., qd×14 d	→ STAT1/IRF1	Hu et al., 2025
	Betulinic acid	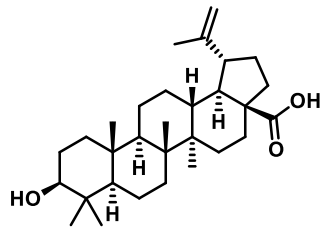	*Betula* spp. (e.g., *Betula platyphylla* Suk.)	*In vitro* H1299, A549, and LLC cell lines,<br>*In vivo* LLC subcutaneous tumor model	*In vitro* 10, 20, 40 μmol/L;<br>*In vivo* 20, 40 mg/kg, i.p. qd×21 d	⊣ mTOR	Zeng et al., 2024
	Triptolide	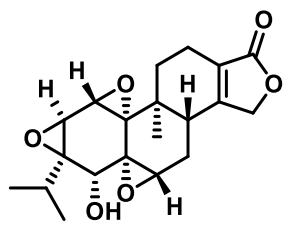	*Tripterygium wilfordii* Hook.f.	*In vitro* RAW264.7 mouse monocyte-macrophage cell line;<br>*In vivo* 4T1 subcutaneous tumor co-implantation model with M2-polarized RAW264.7 macrophages	*In vitro* 5, 10, 20, 40 nM;<br>*In vivo* 0.05, 0.2 mg/kg, i.g. qd×28 d	⊣ CD206, Arg-1, CD204	Li H., et al., 2020
	β-Elemene	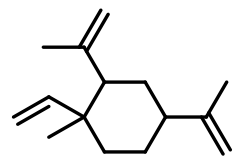	*Artemisia annua* L.	*In vitro* A549 and H1299 cell lines;<br>*In vivo* A549 subcutaneous xenograft cell transplantation tumor model	*In vitro* 2mg/mL<br>*In vivo* not mentioned	⊣ CBX8 → ↑ miR-127-3p → ⊣ MAPK4 → ⊣ Akt/mTOR/p70S6K	Wu, X., et al., 2024
	Scutebarbatine A	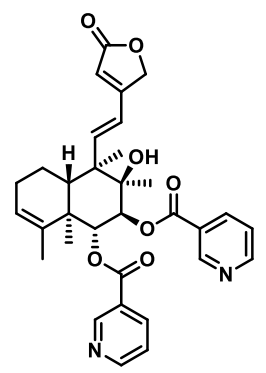	*Scutellaria barbata* D. Don	*In vitro* PMA-differentiated THP-1 macrophages; IL-4/IL-13-induced M2 polarization; A549, H1299 cell lines;<br>*In vivo* Nude mouse xenograft tumor models established with A549 cells alone or A549 cells co-injected with IL-4/IL-13-treated THP-1 cells.	*In vitro* 40 μM;<br>*In vivo* 40 mg/kg, i.g., qd×21 d	⊣ PI3K/Akt → ↓ CD206/Arg-1/PPAR-γ	Zhang, Y., et al., 2026
	Tubeimoside II	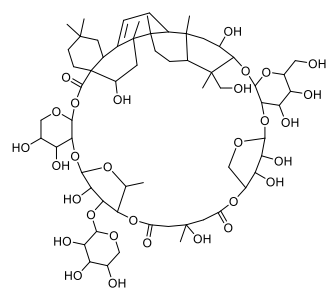	*Bolbostemma paniculatum* (Maxim.) Franquet	*In vitro* A549 and H1299 cell lines;<br>*In vivo* LLC subcutaneous syngeneic cell transplantation tumor model; A549 subcutaneous xenograft cell transplantation tumor model; PDX model derived from NSCLC patients	*In vitro* 5, 10 ,20 μg/mL;<br>*In vivo* 5, 10 mg/kg, i.p., qd×12 d	⊣ HERC2;<br>⊣ JAK2/STAT3	Xu, T., et al., 2026
Polysaccharides	Astragalus Polysaccharides	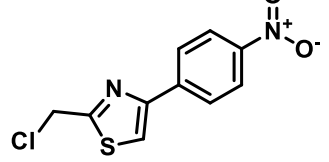	*Astragalus membranaceus* (Fisch.) Bunge	*In vitro* H441、H1299、H1437 cells; PMA-differentiated THP-1 macrophages; IL-4/IL-13-induced M2 polarization;	*In vitro* 8, 16 mg/ml;<br>*In vivo* 3 mg/kg, i.p. qd, ×112 d	→ DC maturation;<br>→ T cell-mediated immunity	Bamodu et al., 2019

				In vivo LLC1 intraperitoneal tumor xenograft model; Intraperitoneal mixed xenograft model co-injected with H1437 and THP-1 cells			
	β-glucan	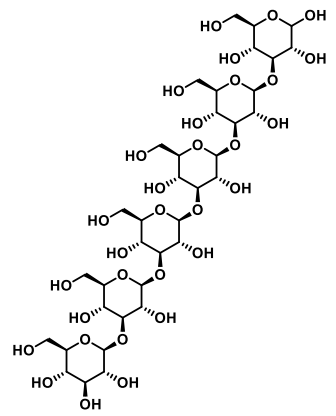	Oats, barley, wheat, baker's yeast cells, fungal cell walls, and certain microorganisms	*In vitro* TAM isolated from LLC tumor-bearing mice; *In vivo* subcutaneous mixed xenograft model co-injected with LLC and TAM cells	*In vitro* 100 mg/ml; *In vivo* 800 mg/mouse, i.g., qd, ×24 d	→ Dectin-1/Syk/CARD9/ERK ; metabolic reprogramming	Liu, M., et al., 2015
	Lentinan	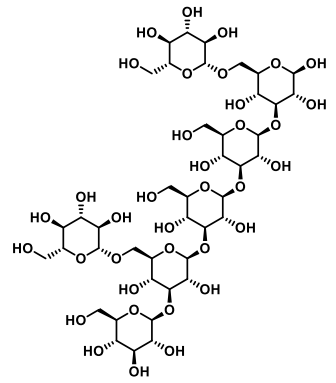	*Lentinus edodes* (Berk.) Sing.	*In vitro* A549, H1299cells; PMA-differentiated THP-1 macrophages; IL-4/IL-13-induced M2 polarization	*In vitro* 2.5, 5 ,10 μM	⊣ Wnt/β-catenin, MMP9	Yang, Y., et al., 2024
Saponins	Ginsenoside Rh2	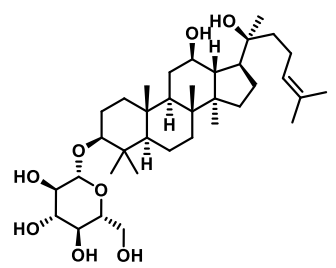	*Panax ginseng* C.A.Mey.	(i) *In vitro* A549, H1299cells; PMA-differentiated THP-1 macrophages; IL-4/IL-13-induced M2 polarization； *In vivo* LLC subcutaneous xenograft mouse model; (ii) *In vitro* A549, H1299, H1975, H1993 cell lines; PMA-differentiated THP-1 macrophages; *In vivo* A549 subcutaneous xenograft cell transplantation tumor mode	(i) *In vitro* 5, 10, 20, 40, 60, 80, 100, 120 μM; *In vivo* 40 mg/kg, i.p., qd ×21 d (ii) *In vitro* 40 μg/mL; *In vivo* 20 mg/kg, i.p., q3d ×40 d	⊣ VEGF, MMP-2/9	(i) Li, H et al., 2018; (ii) Chen, Y., et al., 2026
	Ginsenoside Re	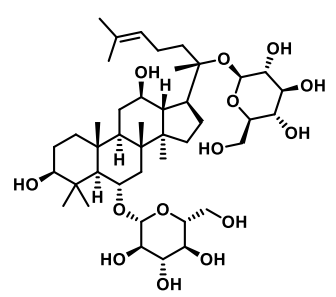	Fruits, leaves, stems, flower buds, and roots of *Panax ginseng* C.A.Mey.	*In vitro* Model of THP-1 monocytes and PMA-induced THP1-DMs macrophages; *In vivo* LLC subcutaneous xenograft mouse model	*In vitro* 0.82, 1.64 μg/mL; *In vivo* 10, 20 mg/kg, i.p. qd × 10 d	⊣ AMPKα1/STING	Tang, X., et al., 2024
	Ginsenoside Rb1	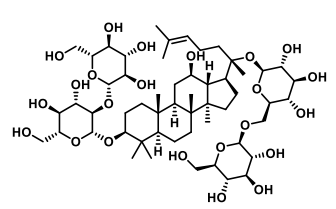	*Panax ginseng* C.A.Mey., *Panax notoginseng* (Burkill) F.H.Chen ex C.Chow	*In vitro* PI3K E545K mutant anti-PD-1 resistant 344SQ cells (344SQ_R); *In vivo* PI3K E545K mutant A549 subcutaneous xenograft nude mouse model; Anti-PD-1 resistant syngeneic NSCLC mouse model bearing 344SQ_R cells	*In vitro* 5 μg/mL; *In vivo* 10 mg/kg, iv, q7d ×3	⊣ PI3K/Akt/mTOR; ⊣ PD-1/PD-L1	Jin, X., et al., 2025

	Astragaloside IV	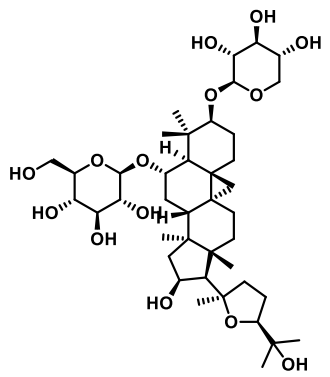	*Astragalus membranaceus* (Fisch.) Bunge	*In vitro* THP-1 M0/M1/M2 macrophages; A549 human lung cancer; H1299 human lung cancer; LLC murine Lewis lung carcinoma;<br>*In vivo* LLC subcutaneous xenograft mouse model；LLC intravenous lung metastasis mouse model	*In vitro* 40/80 μM;<br>*In vivo* 40 mg/kg, i.g., qd ×21 d	⊣ AMPK	Xu, F., et al., 2018
**Stilbenoids**	Resveratrol*	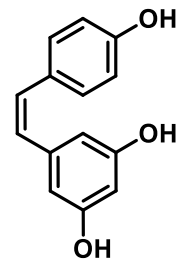	*Vitis vinifera* L., berries, *Arachis hypogaea* L., *Chenopodium quinoa* Willd.	*In vivo* Lewis lung carcinoma mice	*In vivo* 50mg/kg, i.g., qd×21 d	→ G-MDSC apoptosis; → M-MDSC differentiation	~~Y. Zhao, 2018;~~ Zhao, Y., et al., 2019
	Trismethoxyresveratrol	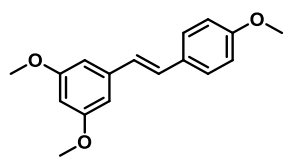	Natural derivative of resveratrol	*In vitro* co-culture model of PMA-differentiated THP-1 macrophages and H1299/H1975 lung cancer cells;<br>*In vivo* H1299 subcutaneous xenograft model in nude mice	*In vitro* 5, 10, 25, 50, 100 μmol/l;<br>*In vivo* 10, 30, 50 mg/kg, i.g., qd×25 d	⊣ circPACRGL/IGF2BP2/YAP1, Hippo	Liu & Xiong, et al., 2024
	Pterostilbene	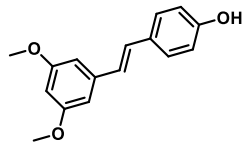	Blueberries	*In vitro* 549 and H411 cell lines; PMA-differentiated THP-1 macrophages; IL-4/IL-13-induced M2 polarization；	*In vitro* 5, 10, 20 μM	⊣ MUC1/NF-κB	Huang, W., et al., 2016
Lignans	Honokiol	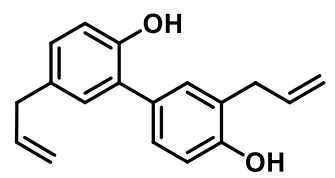	*Magnolia officinalis* Rehder & E.H.Wilson	*In vitro* Human lung cancer A549 and H460 cells; Murine Lewis lung carcinoma LL2 cells;<br>*In vivo* LL2 subcutaneous tumor-bearing mouse model; LL2 lung metastasis mouse model	*In vitro* 10, 20 μg/mL;<br>*In vivo* 20 mg/kg, i.v., q2d, ×20~25 d	⊣ PI3K/Akt	Cheng, Y., et al., 2024
Phenolic acids	Rosmarinic acid	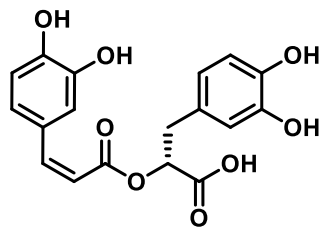	*Rosmarinus officinalis* L., *Rhus chinensis* Mill.	(i) *In vitro* RAW264.7 cells;<br>(ii) *In vitro* HCC827, PC9, A549, H1975 NSCLC cells were co-cultured with MRC-5 human embryonic lung fibroblasts;<br>*In vivo* Single-cell HCC827 NSCLC subcutaneous xenograft model	(i) *In vitro* 0.625, 1.25, 2.5, 5, 10, 20, 40, 80, 160 μmol/L;<br>(ii) *In vitro* 5 μmol;<br>*In vivo* 10 mg/kg, i.g. qd×6/12 d	⊣ NF-κB (p-p65)	(i) Huang Y., et al., 2025;<br>(ii) Li, D., et al., 2025

Note: * Denotes metabolites with a high risk of PAINS, →, activation or promotion; ⊣, inhibition or suppression. Metabolites are grouped by chemical class. Detailed mechanisms and supporting references are described in the corresponding subsections of Section 3.
